# Observation of magnon polarons in the van der Waals itinerant ferromagnet Fe_3_GeTe_2_

**DOI:** 10.1038/s41467-026-74465-3

**Published:** 2026-06-20

**Authors:** Qili Li, Namrata Bansal, Paul Nufer, Lichuan Zhang, Amir-Abbas Haghighirad, Christoph Sürgers, Yuriy Mokrousov, Wulf Wulfhekel

**Affiliations:** 1 Physikalisches Institut, Karlsruhe Institute of Technology, Karlsruhe, Germany; 2 School of Physics and Electronic Engineering, Jiangsu University, Zhenjiang, China; 3 Peter Grünberg Institut (PGI-1) and Institute for Advanced Simulation (IAS-1) Forschungszentrum Jülich GmbH, Jülich, Germany; 4 Institute for Quantum Materials and Technologies, Karlsruhe Institute of Technology, Karlsruhe, Germany; 5 Institute of Physics, Johannes Gutenberg-University Mainz, Mainz, Germany

**Keywords:** Magnetic properties and materials, Two-dimensional materials, Electronic properties and materials, Characterization and analytical techniques

## Abstract

Magnon polarons, hybrid quasiparticles embodying the intrinsic coupling between spin and lattice dynamics, bridge magnetism and phononics in quantum materials. Despite extensive studies in magnetic insulators, spectroscopic evidence for magnon polarons has been scarcely reported in metallic ferromagnets. Here, we reveal magnon polarons at the nanoscale in single crystals of Fe_3_GeTe_2_ using inelastic scanning tunneling spectroscopy (ISTS) with a milli-Kelvin scanning tunneling microscope. While ISTS of phonons or magnons has been widely explored, our study focuses on two-dimensional van der Waals (vdW) itinerant ferromagnets, where momentum selection rules normally hinder magnon excitation. We show that strong magnon-phonon interaction leads to hybridization between the magnonic and phononic bands, lifting the selection rules at their avoided band crossings. This emergent momentum selection due to magnon-phonon coupling is a hallmark of magnon polarons. Our findings establish a platform for probing and engineering magnon-phonon hybrid excitations in two-dimensional materials at the nanoscale.

## Abstract (web summary)

Two-dimensional magnetic materials have magnetic properties that are sensitive to strain, implying a strong coupling between the magnetic and lattice degrees of freedom. Here, using inelastic scanning tunnelling spectroscopy Li, Bansal, and coauthors find the telltale signatures of magnon-polarons in the two-dimensional ferromagnet, Fe_3_GeTe_2_.

## Introduction

The neoteric exploration of two-dimensional (2D) van der Waals (vdW) magnets has recently extended the philosophy of magnonics and spintronics into the 2D realm^1–10^. There, due to their 2D nature, the elementary bosonic excitations in the form of magnons and phonons inherit the strong symmetry breaking of the 2D material, such as anisotropic dispersion^11–15^ and chirality^16,17^. In magnetic 2D materials, the large uniaxial anisotropy and magnetic properties are extremely sensitive to strain engineering^18–23^ owing to the low symmetry with strong crystal fields caused by the local surroundings. This potentially leads to an unusually high dynamic coupling between magnetic and lattice degrees of freedom in two dimensions, with a strong impact on the dynamical properties of 2D magnets. However, very little is known about the nature and properties of elementary magnetic and lattice excitations in 2D magnetic materials. This necessitates the exploration of such excitations, both theoretically and experimentally with various techniques, among which inelastic scanning tunneling spectroscopy (ISTS) emerges as a powerful method^24–30^. ISTS allows us to probe inelastic excitations in structurally complex 2D materials locally^31^. By now, ISTS has developed into a well established technique to locally resolve vibrons in adsorbed molecules^32^, but also has the potential to resolve bulk phonons^29,30^ mostly in materials with strong electron-phonon coupling, i.e., superconductors, surface phonons in noble metals^33–35^, and phonons in 2D materials like graphene^36,37^. Furthermore, ISTS has also been used for the detection of magnons^24–28^.

Among 2D magnets^1,4–10^, Fe_3_GeTe_2_ (FGT) fascinates with its high ferromagnetic transition temperature of 150-220 K in bulk^38–40^, even reaching room temperature with gate tuning^41^, and metallic traits^38,39^ suitable for an investigation by scanning tunneling microscopy (STM)^42–46^. This prolific material serves as a playground for investigating magnetic skyrmion bubbles^43,45,47–50^, spin spirals^51^, magnetic domains with sharp domain walls^45,46,49,50,52^, large uniaxial anisotropies^38–40,53–55^ and various prominent magnetic properties in a 2D world^16,53,55–58^. Furthermore, FGT is predicted to show strong magnetoelastic coupling^18^, and spin-optical phonon coupling has been investigated by Raman spectroscopy^59–61^. However, it is still elusive for ISTS to detect excitations in itinerant ferromagnets with strong magnon-phonon coupling. While magnon polarons have been reported in magnetic insulators, like YIG^62^, Cr_2_O_3_^63^, and 2D FePS_3_^64–67^ via inelastic neutron scattering, spin Seebeck effect or Raman spectroscopy, it is rarely studied in metallic ferromagnets. This makes FGT an ideal system to study magnon polarons due to magnons, phonons and their interactions by ISTS.

Here we report the nanoscale observation of magnon polarons in the 2D ferromagnetic vdW material FGT detected by laterally resolved and spin-polarized ISTS measurements executed with STM under ultrahigh vacuum and at low temperatures (40 mK). Supported by density functional theory and model calculations, we identify the quasiparticle excitations in ISTS of FGT as fingerprints of hybrid magnon-phonon modes (i.e., magnon polarons), which emerge at the crossing points between the magnonic and phononic bands. At these points of strong hybridization, the selection rules for magnon and phonon excitation are softened. This emergent momentum selection largely enhances the scattering cross section with electrons. Our findings provide a platform for investigating and designing the properties of magnon polarons in two-dimensional materials at the nanoscale.

## Results

### Principles of ISTS

**Fig. 1:**
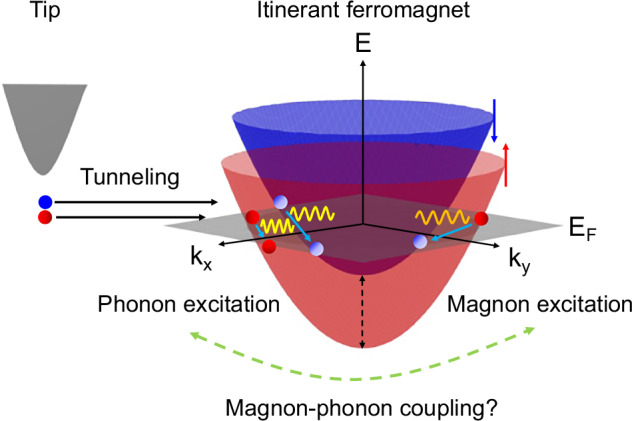
Sketch of magnon and phonon excitations in an itinerant ferromagnet. Phonon excitation (yellow wavy line) is a non-spin-flip process, while magnon excitation (orange wavy line) requires spin flip. Red (blue) indicates spin up (down). The dashed black arrow indicates the exchange splitting (or Stoner gap). A question remains: what will happen if there is magnon-phonon coupling?

In general, ISTS provides information on the scattering cross section of hot carriers with bosonic excitation in the samples. Within ISTS, inelastic excitations show up most clearly in the form of peak-dip pairs in the second derivative of the tunneling current with respect to the bias voltage, symmetrically located in bias voltage around the Fermi level^68^. Vibrons or phonons are created by Coulomb scattering of the hot carriers with the core electrons of the atoms, and thus the cross section does not depend on the spin of the tunneling electron and is typically only of the order of a few percent of elastic scattering. In a 2D sample, the hot carriers injected by the tip thus need to relax by an energy *ΔE* closer to the Fermi energy by exciting a phonon with energy *ℏω* = *ΔE*. As the 2D sample is translational invariant with respect to translation in the 2D plane, the in-plane wave vector **k**_∥_ is a conserved quantity. In the scattering process, the change of wave vector of the electron thus corresponds to the wave vector of the excited phonon. As phonon creation does not involve the spin of the electron, inelastic transitions comprise of transitions between electronic states of the same spin. At low phonon energies, especially for acoustic phonons, inelastic scattering is not prevented by phase space arguments. Arbitrarily small energy transitions are allowed between states of arbitrarily small difference in **k**_∥_ on the same band (see Fig. 1). Thus, one can expect to observe low-energy phonons in ISTS, but with a relatively weak electron-phonon scattering cross-section due to the scattering mechanism. In case of magnons, however, the scattering cross section is significantly larger as the excitation of magnons is driven by the exchange interaction of the tunneling electrons with the spin-polarized sample electrons^25,69–71^. In phase space, the excitations of magnons are coupled to electronic transitions between bands of opposite spin character. The transitions are, however, not possible for combined low momentum and low energy due to the exchange energy (cf. Fig. 1), i.e., the two Fermi surfaces of opposite spin character have usually rather different shapes. This opens a gap for single particle spin-flip excitations, i.e., a Stoner gap, in the spectrum. The Stoner gap suppresses electron-magnon scattering at low energies and momentum such that magnons near the zone center cannot decay into Stoner excitations and thus display long life times. An interesting question remains open: what happens to ISTS if there is magnon-phonon coupling?

When taking into account the coupling of phonons and magnons, these selection rules for inelastic electron scattering are lifted at crossing points of the phonon and magnon dispersion. At these points, a hybridization between phonon and magnon modes leading to magnon-polarons is expected, and both the strong exchange interaction and the core scattering can excite the hybrid boson. This effect should show up in the ISTS spectra as distinct peaks at very specific energies given by the dispersion of magnons and phonons.

### Quasiparticle excitations

**Fig. 2:**
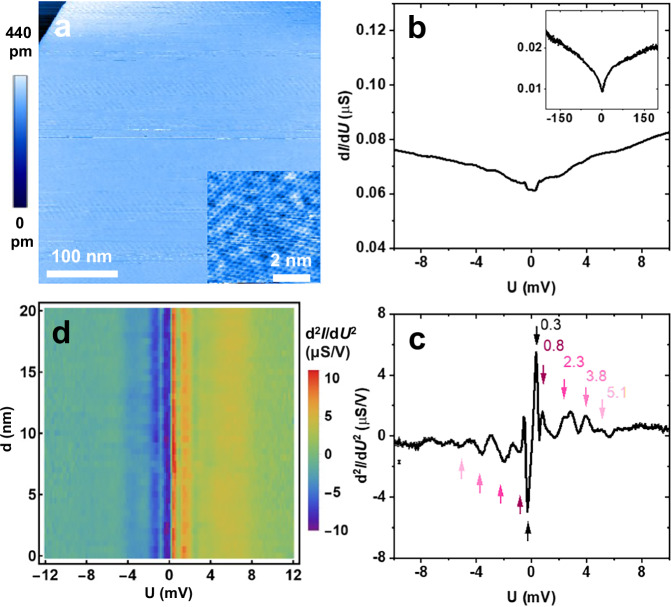
Low energy excitations via ISTS. **a** Topography of the FGT surface. The inset shows the hexagonal lattice, which is the uppermost Te atoms. **b** *dI*/*dU* spectrum averaged over 121 individual curves in a 4 × 4 nm^2^ area. The spectra were measured with a W tip at 40 mK (feedback conditions *U* = 20 mV, *I* = 2 nA, ${U}_{{{{\rm{mod}}}}}=0.5$ mV at 3.421 kHz), and the inset figure in (**b**) was measured with *U* = 50 mV, *I* = 1 nA and ${U}_{{{{\rm{mod}}}}}=3$ mV at 3.611 kHz. **c** *d*^2^*I*/*dU*^2^ spectra recorded simultaneously in (**b**). Arrows mark the peak-dip pairs, and error bars show the standard deviation of the mean. **d** ISTS spectra recorded along a line of 20 nm (*U* = 20 mV, *I* = 5 nA modulation amplitude ${U}_{{{{\rm{mod}}}}}=0.5$ mV at 3.611 kHz).

In FGT, it is widely reported that there is a broad dip in the differential conductivity *dI*/*dU* near the Fermi energy (*E*_*F*_)^42,45,46^, as depicted in the inset of Fig. 2b. The dip has been interpreted by Zhang et al. as a Kondo resonance^42,72^, which is disputed in the literature^45,46^, as the sample is a ferromagnet with relatively large exchange splitting of the electronic bands^18^. More specifically, the survival of Kondo screening depends on the competition between the Kondo energy scale *E*_*K*_ = *k*_*B*_*T*_*K*_ and the exchange splitting *E*_*ex*_ in a ferromagnetic Kondo lattice. When *E*_*ex*_ ≫ *E*_*K*_, Kondo screening is destroyed. Consistent with this principle, experimentally established ferromagnetic Kondo lattice systems typically satisfy *T*_*K *_≥ *T*_*C*_^73,74^, ensuring that Kondo correlations remain robust against magnetic ordering. Moreover, the spectroscopic feature manifests as a dip rather than a peak. Although such a dip could in principle be rationalized by interference effects within the Kondo framework^75^, a growing body of evidence indicates that many of these Kondo-like dips observed on magnetic adatoms on non-magnetic surfaces are, in fact, signatures of inelastic spinon-polaron (spinaron) excitations^76–78^. A second explanation for the dip in *dI*/*dU* could be a dip in the electronic density of states (DOS). DFT calculations for the DOS of FGT, however, exclude this^45^. A third explanation is the disorder-induced electron-electron interaction^79^. However, our samples have low impurity density (Fig. 2a). A fourth possibility for the dip is magnetic inelastic excitations, which we investigate in the following^80–83^.

In elastic tunneling, the differential conductance *dI*/*dU* is proportional to the DOS of the electrodes^84^. When variations in DOS are negligible on a small energy range around *E*_*F*_, *dI*/*dU* is constant, and *d*^2^*I*/*dU*^2^ vanishes. An additional tunneling channel opens when the kinetic energy of the tunneling electrons is sufficient to cause an inelastic excitation. As the two channels do not interfere, an increment in the current is observed for both signs of the bias voltage. Consequently, steps appear in *dI*/*dU* at the brink of the excitation energy *E*_*g*_ = ∣*eU*∣, or equivalently peaks and dips appear in *d*^2^*I*/*dU*^2^ at ∣*eU*∣ = *E*_*g*_. Fig. 2b shows the differential conductivity in a small interval around *E*_*F*_ recorded at 40 mK with a small lock-in modulation voltage amplitude ${U}_{{{{\rm{mod}}}}}$= 500 μV (root mean square ${U}_{{{{\rm{mod}}}}}^{rms}={U}_{{{{\rm{mod}}}}}/\sqrt{2}$). ${U}_{{{{\rm{mod}}}}}$ was chosen as a compromise of energy resolution and signal-to-noise ratio. Clearly, the spectrum deviates from that of a Kondo resonance as steps at various energies can be resolved. In agreement with this, the *d*^2^*I*/*dU*^2^ spectra, recorded simultaneously with the *dI*/*dU* spectra, show five clearly resolved peak-dip pairs symmetrically positioned with respect to *E*_*F*_, cf. Fig. 2c. The error bars in the plot represent the 1*σ* error of the averaged signal and document the significance of the peaks. Hence, the data in Fig. 2b, c are clear evidence for the occurrence of inelastic tunneling events. To illustrate the homogeneity of the observed inelastic signal over longer distances, we show the variation of the ISTS signal over 20 nm in Fig. 2d. The peak-dip pairs only show a slight intensity variation within the noise level. This homogeneity reflects the high quality and low Fe deficiency of our samples. We note that our measurements were performed at different sample positions and several samples with different tips, yielding consistent spectral features and demonstrating the excitations’ intrinsic nature.

At this low-energy scale of the peaks of 0.3, 0.8, 2.5, 3.8 and 5.2 meV with an experimental resolution of  ± 0.3 meV (see arrows in Fig. 2c), these intrinsic inelastic excitations can be due to either phonons or magnons near the $\overline{\Gamma }$ point, while plasmons, with eV energy, can be excluded. However, note that the individual pairs of peaks and dips are not identical in intensity. Typically, vibron or phonon excitations are rather symmetric in intensity. For magnons, strong asymmetries have been reported due to spin selection rules^25^. To create a magnon by inelastic exchange scattering, either a minority electron needs to be injected into the ferromagnet, or a majority electron needs to be removed. Thus, the asymmetry in intensity of magnon excitation peaks relates to the spin polarization of the magnetic sample, in the case an unpolarized tip is used. The calculated asymmetry, i.e., the difference in the peak area for negative and positive bias divided by their sum, taken from the data shown in Fig.2c, is about  − 0.1, which is comparable to a DFT value for spin-polarization of the states of about  − 0.36 at the Fermi energy^45^. This suggests a magnetic origin of these excitations. Moreover, the ISTS spectra strongly change with magnetic field i.e., the energy peak at 0.8 meV shifts upward to 1.1 meV at 3 T and 1.25 meV at 5 T, in quantitative agreement with the expected Zeeman energy shifts of 0.35 meV (3 T) and 0.58 meV (5 T), while the higher-energy peaks shift downward with increasing magnetic field (see Supplementary Fig. 1). This excludes phonons as sole origin and proves a magnetic component of these excitations in FGT. Note that the peak at 0.3 meV remains unchanged under an applied magnetic field. In particular, it consistently appears at very low temperatures in many samples and has been attributed to a dynamical Coulomb blockade effect^29,85–87^. This effect comes from the diverging lifetimes of quasiparticles in Fermi liquids near the Fermi energy and at low temperatures, hindering a second low-energy electron to tunnel into the same state until the first is not scattered. It has also been seen on noble metals at low temperatures^87^ and manifests itself as a dip at zero bias in *dI*/*dU* of a width of about twice ${U}_{{{{\rm{mod}}}}}^{rms}$ or as a peak-dip feature in *d*^2^*I*/*dU*^2^ at $\approx \pm {U}_{{{{\rm{mod}}}}}^{rms}$. Note that a higher temperature will smear and broaden these peak-dip pairs into one broadened peak (see Supplementary Fig. 5). Similarly, a larger modulation voltage also results in a rather broad peak-dip pair at energy around 6 meV and beyond. However, in this work, we intend to focus on the sharp inelastic peaks and dips of low energy.

### Spin-polarized results

To further reveal the origin of these excitations, ISTS with a spin-polarized (SP) tip was employed. In this technique, the STM tip is spin-polarized, such that the tunneling magneto-resistance effect (TMR)^88^ arises, providing spin-resolved information on the sample. To polarize the tip,  ≈ 50 ML Cr was deposited on freshly prepared W tips, followed by gentle annealing^89^. The use of antiferromagnetic tips minimizes the magnetic stray field, which exerts a negligible influence on the magnetization of the FGT sample^45^. This allows us to compare inelastic tunneling spectra for the relative parallel and antiparallel orientation of the magnetization of the electrodes, which is the standard test for magnetic excitations^24,90^.

**Fig. 3:**
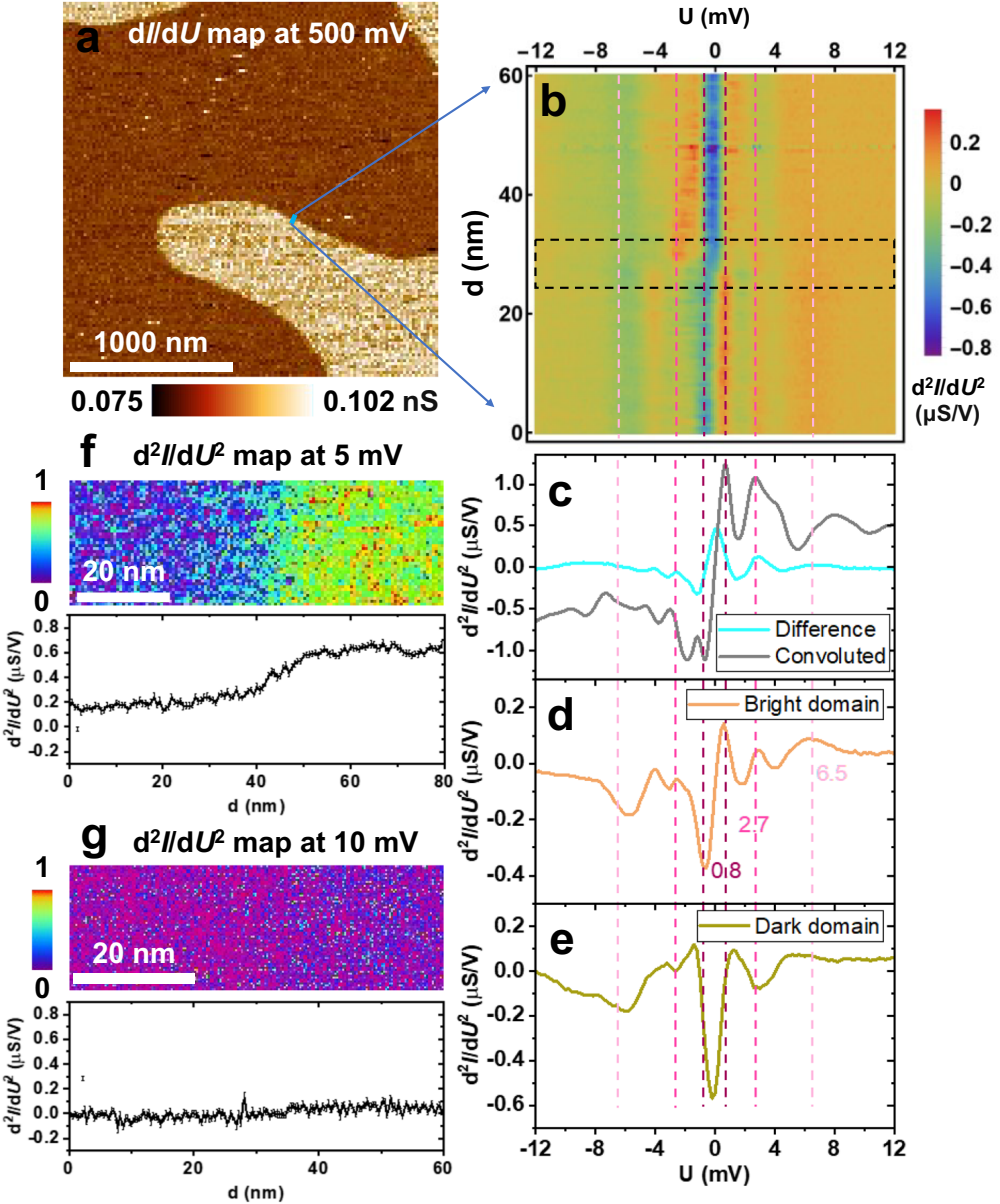
Spin-polarized results. **a** *dI*/*dU* map at 500 mV with Cr-coated W tip (tunneling condition *U* = 500 mV, *I* = 0.1 nA, and ${U}_{{{{\rm{mod}}}}}^{rms}=100$ mV). **b** SP-ISTS spectra across a magnetic domain wall (*U* = 20 mV, *I* = 0.3 nA, and ${U}_{{{{\rm{mod}}}}}=$1 mV). The dashed rectangle indicates the domain wall. **c** The modulation convoluted ISTS of Fig. 2**c** (gray) and the difference of *d*^2^*I*/*dU*^2^ spectra (cyan) between bright domain (**d**) and dark domain (**e**). The vertical dashed lines indicate the peak-dip pairs. **f** *d*^2^*I*/*dU*^2^ map at 5 mV and the average line profile (*U* = 5 mV, *I* = 0.2 nA, and ${U}_{{{{\rm{mod}}}}}=2$ mV at 3.611 kHz). **g** *d*^2^*I*/*dU*^2^ map at 10 mV and the average line profile (*U* = 10 mV, *I* = 0.3 nA, and ${U}_{{{{\rm{mod}}}}}=2$ mV at 3.611 kHz).

In the following, we compare the ISTS spectra recorded along line scans using unpolarized W tips and Cr-coated tips with out-of-plane spin polarization^45^. To confirm the spin polarization of the tip, we measured *dI*/*dU* maps at 500 mV to find magnetic domains (Fig. 3a). As shown in Fig. 3a, we observe a high spin contrast at 500 mV of about 15%. Then, we zoomed in to measure the low-energy excitations by ISTS across domain walls. Similar to the unpolarized results shown in Figs. 2, 3b displays the position-dependent but now spin-polarized inelastic tunneling signal across the magnetic domain wall. To improve the signal-to-noise ratio, a larger modulation voltage was used. Clearly, a lateral variation in the inelastic signal, which coincides with a 180^∘^ magnetic domain wall in SP-STM *dI*/*dU* map (Fig. 3a) can be seen. Thus, on opposite sides of the domain wall, the relative orientation between the tip and sample magnetization changes by 180^∘^ and the relative role of minority and majority carriers in the sample is exchanged. The dashed rectangle in Fig. 3b indicates the domain wall. Figure 3c shows the difference of *d*^2^*I*/*dU*^2^ spectra between bright domain (Fig. 3d) and dark domain (Fig. 3e), i.e., the spin-polarized ISTS signal. For comparison, the unpolarized ISTS signal of Fig. 2c has been reproduced, including a convolution taking account of the different modulation voltages (see Supplementary Information Note III). Energies representing pairs of peaks and dips in the spectra are indicated by dashed lines. We find back the 0.8 meV excitation in all spectra, especially in the difference signal. The peak structures near 2.3 meV are slight shifted upward to 2.7 meV in difference and the convoluted spectrum. The peak at 5.2 meV together with a broad peak/dip structure above it becomes more visible in the difference and convoluted signal as a broad peak at 6.5 meV. All three peak-dip pairs at 0.8, 2.7 and 6.5 meV can be seen for the difference of SP-ISTS on bright domain and dark domain, which agrees well with the convoluted ISTS (see gray curve in Fig. 3c). To further showcase this transition across the domain wall, the *d*^2^*I*/*dU*^2^ map at 5 mV, i.e., the third peak in Fig. 3b–e was measured. Figure 3f exhibits an obvious transition from a dark domain to a bright domain, where the change of contrast reaches 200%. As a comparison, a *d*^2^*I*/*dU*^2^ map at 10 mV is shown in Fig. 3g, where this transition is not observed. Note that the spin-polarization of the tip was checked, followed by repeating the *d*^2^*I*/*dU*^2^ map at 5 mV. Overall, the behavior of the signal across the domain wall is in agreement with the spin selection rules for magnon excitation^25^.

**Fig. 4:**
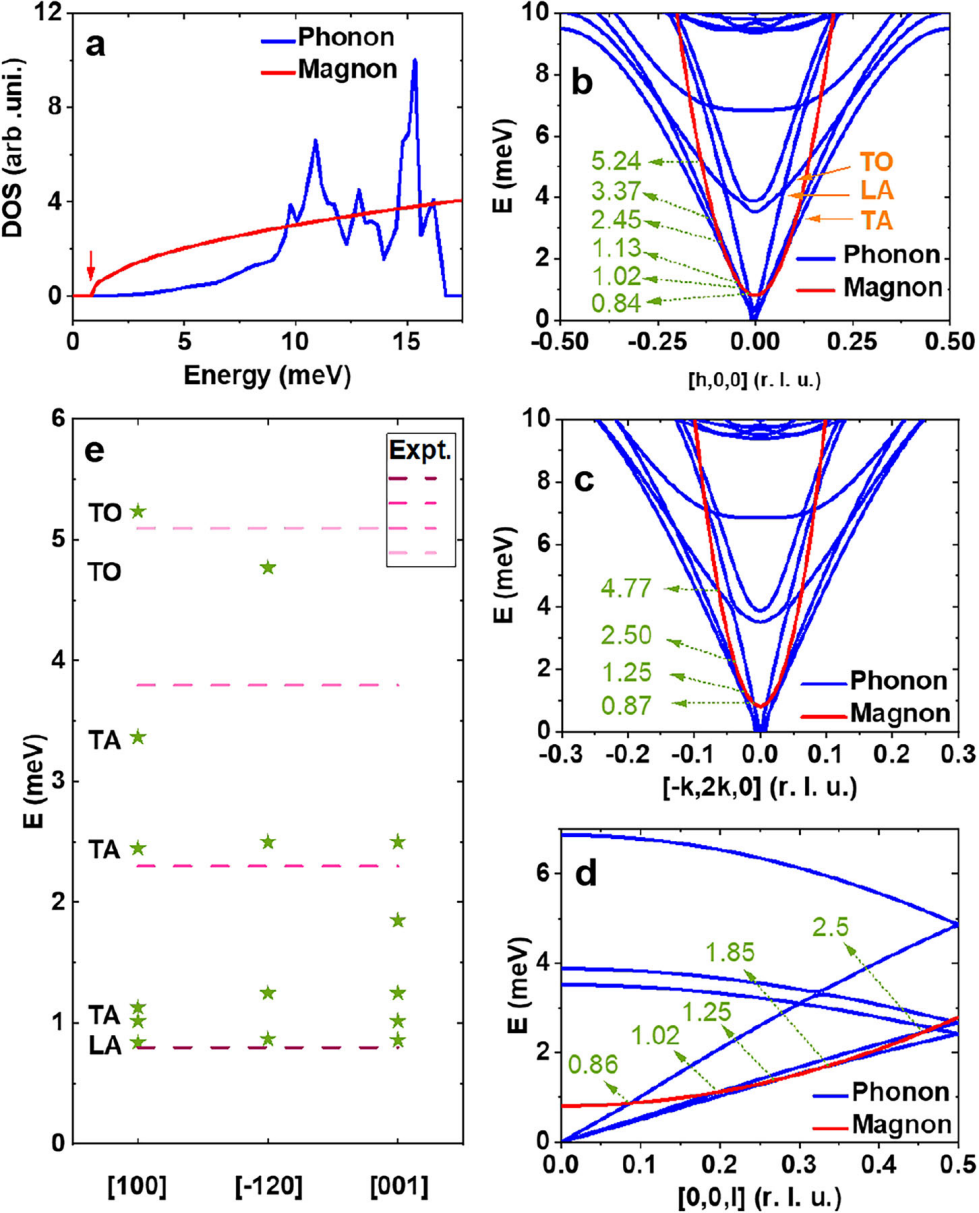
Magnon and phonon band crossings in FGT. **a** Magnon and phonon DOS in FGT. The phonon DOS is calculated from DFT. The magnon DOS is calculated from three-dimensional, anisotropic and parabolic dispersions around the Brillouin zone center. The red arrow indicates the spin-wave magnon gap (0.81 meV) for our samples. **b** Band crossings between magnon and phonon bands along [100] in reciprocal lattice unit (r.l.u.). The transverse acoustic (TA), longitudinal acoustic (LA), and transverse optical (TO) phonon modes are indicated by the orange arrows. **c** Crossings between magnon and phonon bands along $[\overline{1}20]$. **d** Crossings between magnon and phonon bands along [001]. The spin-wave stiffness for [100], [$\bar{1}$20], and [001] are 69.0 meVÅ^2^, 56.7 meVÅ^2^, and 53.6 meVÅ^2^ from ref. ^14^. **e** Magnon-phonon band crossings (stars) compared with *d*^2^*I*/*dU*^2^ peaks (dashed lines).

This identifies the peaks as magnetic excitations (or magnons) but does not provide an explanation for their specific energies. Note that the parabolic dispersion of magnons around the zone center does not produce a peaked DOS as shown in Fig. 4a and Methods. Moreover, at low energies, magnon excitation in the 2D vdW material FGT is expected to be suppressed due to the exchange splitting of the bands, i.e., the Stoner gap.

Phonons might lead to sharp van Hove singularities in the meV range, as ab-initio calculations of the phonon DOS, shown in Fig. 4a in blue, indicate. The peaks in the DOS are between 10 and 15 meV, which are consistent with the results of inelastic neutron scattering^14,15^. Note that we did not observe these phonon peaks in the ISTS shown in Fig. 2. This implies a weak electron-phonon coupling in agreement with recent estimates of only a small electron-phonon coupling constant^91^. Below 5 meV, the phonon DOS is smooth as the lowest energy bands for transversal and longitudinal acoustic phonons disperse linearly in this energy range. Thus, there are no van Hove singularities in the phononic DOS below 5 meV (Fig. 4a), where the ISTS peaks were observed, excluding bare phonons as their origin. The same argument holds for bare magnons, also showing no van Hove singularities in the energy range. When allowing for magnon-phonon coupling, however, the situation drastically changes, as described in the introduction. In case of a crossing between the phonon and magnon bands, magnetoelastic coupling (*H*_*me*_ = ∑_*ij*_*B*_*ij*_*ϵ*_*ij*_*S*_*i*_*S*_*j*_, with *B*_*ij*_ the magnetoelastic coupling constants, *ϵ*_*ij*_ the strain tensor, and *S*_*i*_ the spin component^92^) hybridizes the two distinct modes and an avoided level crossing is expected. At these so-called “hot spots" in the Brillouin zone, magnetization strongly couples to the lattice, which is also responsible for parts of Gilbert damping^93^.

### Magnon and phonon band crossings

We suggest that in FGT, such avoided level crossings with diabolic points cause a strong coupling of electrons to the hybrid magnon polarons. In Fig. 4b we plot the ab-initio phonon dispersion of FGT together with an experimental dispersion curve of the magnons obtained from neutron scattering^14^, which currently provides the most reliable and quantitative information on the magnetic excitation spectrum of FGT. Note that our samples are of higher quality than those of the neutron scattering experiments, which support the use of the applicability of the experimental magnon dispersion to our work. The measured spin-wave stiffness from neutron scattering is about 69.0 meVÅ^2^ along the [100]-direction, whereas the calculated spin-wave gap or anisotropy induced magnon gap at *Γ* point is 0.81 meV for our samples (see “Methods”). This gap agrees well with the recent experimental determination of the gap in FGT of similar quality^94^. Thus, the plot does not contain any fitting parameters. The plot predicts band crossings of the acoustic phonons – with their linear dispersion – with the parabolic but gapped magnon spectrum very close to the $\overline{\Gamma }$-point at 0.9 meV along all directions in agreement with the observed peak and dip in the inelastic spectrum (see Fig. 4b–d for band crossings in three directions and Supplementary Fig. 6 for identifying magnon-phonon band crossings). As the quadratic magnon dispersion rises eventually faster, more crossings may appear along the different directions. Note that the magnon dispersion is anisotropic and in the [001]-direction, i.e., normal to the vdW layers, the exchange is weak so that a second crossing with all except the lowest phonon band can be excluded (see Fig. 4d). Figure 4e shows magnon-phonon band crossings compared with *d*^2^*I*/*dU*^2^ peaks. Along the high-symmetry [100]-direction, second crossings are expected at 2.3, 3.6 and 5.1 meV in excellent agreement with the experimental observations. Crossings along the lower symmetry $[\overline{1}20]$-direction are expected to have a lower spectral weight, and the corresponding peaks are not clearly resolved, or no hybridization occurs in these directions. The theoretically predicted crossing points are in very good agreement with the position of the experimental inelastic peaks and dips shown in Fig. 2d, which clearly demonstrates for the first time the presence of strong magnon-phonon coupling in one of the most prolific 2D materials. Note that there are no crossings below the spin-wave gap 0.81 meV. Interestingly, optical phonons also cross the magnon dispersion at higher energies, where neutron scattering data show that the magnon dispersion becomes heavily damped^13–15^. At these energies, no sharp van Hove singularities are expected, but magnon-phonon coupling for optical phonons has been detected using Raman spectroscopy^59,61^.

### Model calculations for magnon-polaron excitations

To model our experimental results, we develop a theory of ISTS for magnon-polaron excitation by tunneling electrons (see “Methods”), which is beyond the usual ISTS theory widely applied for phonons and magnons. In ISTS, bosons are excited by tunneling electrons, which is distinct from inelastic neutron scattering^95^ and Raman spectroscopy^96^, as the electrons themselves deliver the magnetism. This calls for a quantum field description of the problem. The electrons in a material cannot be seen as naked particles, but get dressed by their coupling to bosons. For electrons coupled to phonons, this has been well described by Engelsberg and Schrieffer^97^. The Green’s function of the dressed electron is given by the Green’s function of the bare electron with its poles in the imaginary part at energies and momenta that correspond to the electron dispersion that becomes lifetime broadened and obtains side bands or kinks due to the phonon energies and momenta^97^. These are the self-energy corrections of the coupled system. Thus, the inelastic tunneling of bare electrons can be regarded as equivalent to the elastic tunneling of phonon-dressed electrons. In summary, ISTS can be understood equivalently as an inelastic scattering process mediated by electron-boson interactions or as an elastic process involving phonon- or boson-dressed electrons. In the method section, the derivation of the latter viewpoint is given in all detail. It turns out that the ISTS signal *d*^2^*I*/*dU*^2^ is related to the imaginary part *ℑ* of the self-energy *Σ*(*ϵ*) in the following way:

1 $$\frac{{d}^{2}I}{d{U}^{2}}\propto -\frac{\partial \Im \Sigma (\epsilon )}{\partial \epsilon }$$

**Fig. 5:**
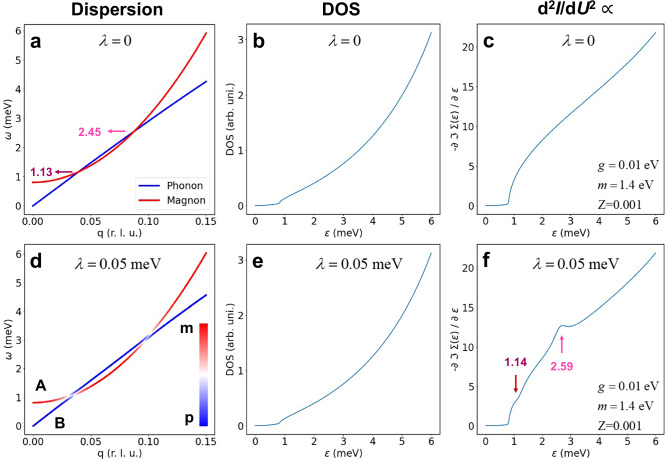
Model calculations with and without magnon-phonon coupling. **a** Band crossings for magnon and phonon dispersions without magnon-phonon coupling. Arrows mark the band crossings. **b** The corresponding DOS for bosons. **c** The derivative of the imaginary part of electron self-energy, i.e., ISTS for magnon polaron. **d** Avoided band crossings for magnon and phonon dispersions with magnon-phonon coupling. **e** The corresponding DOS for magnon polarons. **f** The derivative of the imaginary part of electron self-energy, i.e., ISTS for magnon polaron. Arrows indicate the peaks.

Figure 5a shows the dispersion of the uncoupled bosons and their crossing points, and Fig. 5b shows the sum of their DOS. Here, the magnetoelastic coupling *λ* was set to vanish. As expected, the combined DOS does not show any van Hove singularities in the energy range below 5 meV. While the phonons have no gap at the zone center, the magnons display the usual gap due to magnetic anisotropy. Figure 5c shows the negative derivative of electron self-energy. Without the coupling of magnons and phonons, the calculated ISTS signal does not show peaks. The situation drastically changes when the magnetoelastic coupling is switched on, and the two bosons must hybridize. As expected, the interaction leads to hybridization near the band crossings, as shown in Fig. 5d. While this does not influence the DOS of the bosons (compare Fig. 5b, e) and thus rules out that the observed peaks in the inelastic tunneling spectra are caused by the DOS, the hybridization lifts the spin-selection rules in inelastic scattering for magnon polarons (see “Method” section). As a consequence, the inelastic scattering cross section is enhanced at the avoided crossings Fig. 5f and clear peaks evolve at the energy of the avoided crossings. Outside the peak, inelastic scattering is possible only for phonons, as magnon excitation is prohibited by the Stoner gap. For the optical phonon case, see Supplementary Fig. 7.

Thus, each avoided band crossing manifests as a peak in the derivative of the imaginary part of electron self-energy, namely a peak in ISTS. Note that generally the acoustic phonon bands have two band crossings with the magnon band (Fig. 5), while optical phonon bands have one crossing with the magnon band (Supplementary Fig. 7). According to the magnon-phonon hybridization scenario (Supplementary Figs. 2–4), different hybrid branches exhibit distinct magnetic-field responses: the first avoided crossing with acoustic phonons shifts to higher energies with increasing magnetic field, whereas the second avoided crossing with acoustic phonons and the avoided crossing with optical phonons shifting to lower energies. Thus, the hybrid scenario well explains both the upward shift for the lowest-energy peak at 0.8 meV and the downward shifts of high-energy peaks observed in the experiment (see Supplementary Fig. 1). This also excludes any pure magnon origin, as this would lead only to upward shifts by the Zeeman energy. A further magnon-polaron band-resolved analysis, however, needs the full band structures of electrons, phonon and magnon, and the momentum dependent coupling constants, which is currently beyond the capabilities of DFT. Finally, the characteristic decrease of the ISTS peak intensity and increase in broadening with energy (see Fig. 2c) is in agreement with quasiparticle lifetime effects of electronic nature. Both within Fermi liquid theory and Gilbert damped magnetization dynamics, the lifetime of the excitations decreases with energy, leading to lower peak intensities and broader peaks, as has been reported before^26,27^.

## Discussion

We demonstrate the first nanoscale observation of magnon polarons in the metallic ferromagnet FGT using ISTS, spin-polarized ISTS, magnetic-field and temperature-dependent measurements together with a microscopic model for magnon-phonon hybridization. These hybrid excitations emerge near the Brillouin-zone center at the crossings of magnon and phonon dispersions, exhibiting spin polarization and a distinctive magnetic-field dependence that distinguishes them from a simple coexistence of uncoupled phonons and magnons. The coupling of these hybrid excitations to the conduction electrons is particularly strong in two-dimensional systems, and the suggested mechanism should be present in any magnetic vdW material, as long as the phonon and magnon dispersions cross. Owing to the inherently local nature of ISTS, it also provides a powerful platform for probing confined magnon-polaron modes in these structured samples.

Our findings open venues not only for magnon-phonon engineering but also for exploring fundamental spin-lattice phenomena.

### Magnon-phonon Interconversion

Owing to magnon-phonon coupling, phonons can be used to magnetically excite the system at avoided level crossings. Ultimately, the hybrid bosonic excitations can lead to a chiral nature of the phonons part with the possibility to guide transport of the angular momentum of the lattice. One can envisage that strain engineering could be employed to spatially localize magnons in bulk or even monolayer FGT, enabling magnon waveguides, or inversely, that magnetic domain walls can be used to confine phonons.

### Magnon-polaron damping

Due to the additional electronic degree of freedom inherent to itinerant ferromagnets, we can expect a significant damping of meV magnon polarons near the band crossings. The magnon polarons can decay into phonons, magnons or Stoner pairs of conduction electrons near the hot spots in the dispersion. Consequently, this effect should be experimentally observable in reduced lifetimes of phonons or magnons and a change of electric resistivity (Supplementary Fig. 8^42,98,99^) in the energy and momentum range of the crossings.

### Angular momentum dynamics by magnon polarons

The formation of magnon polarons has previously been discussed in terms of spin angular momentum conservation. However, all three participants of the inelastic process – phonons, magnons and electrons – naturally exhibit orbital magnetism in addition to spin. The processes and corresponding experimental manifestations of spin and orbital angular momentum transfer, conservation, and dynamics among the three degrees of freedom at the hybridization points should be explored in the future. Finally, the hybrid bosonic excitations can lead to a chiral nature of the phonons part with the possibility to guide transport of the angular momentum of the lattice.

## Methods

### Experimental details

High-quality single crystals Fe_3−*x*_GeTe_2_ (*x* ≈ 0.04) were growth by the chemical vapor transport method, which have a Curie temperature *T*_*C*_ ≈ 205 K (see Supplementary Fig. 5 for resistivity and ref. ^45^ for magnetic susceptibility of our samples). Samples were cleaved at room temperature in ultra-high vacuum (UHV) using Kapton tape under a base pressure of *p* < 1 × 10^−10^ mbar, and were immediately transferred to the STM head. This leads to atomically flat surfaces with only very few step edges separated on the micrometer scale [Fig. 2a]. STM measurements were carried out using a home-built low-temperature (40 mK base temperature with variations below 3 mK) dilution STM system^100^.

### Bandstructure and DOS of phonons

To treat the effect of phonons, we calculate their DOS and dispersion in FGT from ab initio using the Vienna ab initio simulation package (VASP)^101^ within the local density approximation (LDA)^102^ for the exchange correlation potential. The projector-augmented wave (PAW) method^103^ is used to describe the interaction between the electrons and the nuclei, and 600 eV was selected for the cutoff kinetic energy of the plane wave expansion. The energy convergence threshold was chosen as 10^−9^ eV and the Brillouin zone (BZ) was sampled with a 15 × 15 × 5 *Γ*-centered Monkhorst-Pack grid^104^. The shape and volume of each cell were fully optimized, and the maximum force in each atom was less than 0.001 eV/Å. The dispersion of phonons was calculated with the code PHONOPY^105,106^ in a 3 × 3 × 2 supercell with the finite displacement method.

### Dispersion and DOS of magnons

The strong unidirectional magnetic anisotropy in Fe_3_GeTe_2_ results in a spin-wave gap or magnon gap. The spin-wave gap was obtained by Kittel’s formula *E*_*g*_ = *gμ*_*B*_(2*K*_*u*_/*M*_*s*_ − *μ*_0_*M*_*s*_) ^107–109^. Here, *K*_*u*_ = 1.46 × 10^6 ^J/m is the unidirectional magnetic anisotropy, and *M*_*s*_ = 3.9 × 10^5^ A/m is the saturation magnetization for our samples^45^. Thus, the spin-wave gap obtained is 0.81 meV, which agrees with the work of ref. ^94^. We note that a smaller spin-wave gap around 0.5 meV measured by inelastic neutron scattering is due to the higher Fe deficiency of their Fe_2.72_GeTe_2_ samples^14^, which also results in a lower T_*C *_= 160 K^14,110^. However, spin-wave stiffness does not show an obvious dependence on Fe concentration^110^. Therefore, we used the spin-wave stiffness determined from inelastic neutron scattering^14^ for magnon dispersion in our sample. The magnon dispersion for Fe_3_GeTe_2_ in the low energy range can be expressed as $E={E}_{g}+{D}_{1}{q}_{1}^{2}+{D}_{2}{q}_{2}^{2}+{D}_{3}{q}_{3}^{2}$, where *D*_*i*_ is the spin-wave stiffness for different directions. Therefore, in the low-energy range, the magnon density of states (DOS) is $N(E)=\Re \frac{\sqrt{E-{E}_{g}}}{4{\pi }^{2}\sqrt{{D}_{1}{D}_{2}{D}_{3}}}$ with *D*_*i*_ constant, where *ℜ* is the real part. The DOS of the magnon increases at a square root rate with energy, while keeping at 0 when E ≤*E*_*g*_.

### Model calculations

#### ISTS for quasiparticle excitations

In the following, we introduce the Hamiltonian for scanning tunneling microscopy, which consists of contributions from the tip, the sample, and their hybridization as below:

2 $$H={H}_{tip}+{H}_{sample}+{H}_{tunnel},$$

where *H*_*tip*_ is the tip Hamiltonian, *H*_*sample*_ is the sample Hamiltonian, and:

3 $${H}_{tunnel}={\sum }_{p,k,\sigma }{W}_{pk}{c}_{p\sigma }^{{{\dagger}} }{c}_{k\sigma }+H.c.$$

is the hybridization between tip and sample.

The STM tunneling current is

4 $$\begin{array}{r}I=\frac{8\pi e}{\hslash }\int \,d\varepsilon {\sum }_{p,k}| {W}_{pk}^{\sigma \sigma }{| }^{2}[ \; f(\varepsilon )-f(\varepsilon -eU)]\\ \times {N}_{p}(\varepsilon )\Im {{{\rm{Tr}}}}({G}^{\sigma \sigma }(k,\varepsilon )),\end{array}$$

where *W*_*pk*_ is the tunneling matrix element, and $\Im {{{\rm{Tr}}}}({G}^{\sigma \sigma }(k,E))$ is the imaginary part of the trace of the spin-resolved retarded Green function of the sample^35,84,111,112^. In general, the tip DOS and tunneling matrix elements do not change much in the low-energy range, and are usually treated as constants.

The measured *d*^2^*I*/*dU*^2^ signal is

5 $$\frac{{d}^{2}I}{d{U}^{2}}(U)=-\frac{8\pi e}{\hslash }{\sum }_{p,k}| {W}_{pk}{| }^{2}{N}_{p}(\varepsilon ){\left[\frac{\partial }{\partial \varepsilon }\Im {{{\rm{Tr}}}}({G}^{\sigma \sigma }(k,\varepsilon ))\right]}_{\varepsilon=eU}.$$

The signal is proportional to the imaginary part of the Green’s function. The retarded Green’s function *G*(*k*, *E*) of the sample can be calculated using the Dyson equation. Expanding the Dyson equation in powers of the self-energy to the lowest order, i.e., *G*(*k*, *ε*) = *G*^0^(*k*, *ε*) + *G*^0^(*k*, *ε*)*Σ*(*k*, *ε*)*G*^0^(*k*, *ε*), where *G*^0^(*k*, *ε*) is the Green function of the bare electron, typically viewed as responsible for the elastic tunneling current. Note that the DOS $N(\varepsilon )=-\frac{1}{\pi }\Im {G}^{0}(\varepsilon )$. The simplified *d*^2^*I*/*dU*^2^^35,113,114^ is below

6 $$\frac{{d}^{2}I}{d{U}^{2}}(U)\approx {\sum }_{p,k}| {W}_{pk}{| }^{2}{N}_{p}(\varepsilon ){N}_{k}{({\varepsilon }_{F})}^{2}{\left[-\frac{\partial }{\partial \varepsilon }\Im \Sigma (k,\varepsilon )\right]}_{\varepsilon=eU}.$$

The above equation indicates that ISTS is proportional to the derivative of electron self-energy. In comparison with ISTS, we can calculate the derivative of the self-energy due to electron-boson interaction.

For electron-phonon interaction, the spin-resolved electron self-energy according to Migdal theorem^97,115–118^ is

7 $${\Sigma }_{ep}^{\sigma }(\varepsilon )={\sum }_{k,q}\int \,\frac{d\omega }{2\pi }| {g}_{k,q}{| }^{2}{G}^{\sigma }(k-q,\varepsilon -\omega )D(q,\omega ),$$

with *g*_*k*,*q*_ is the element of the coupling matrix for the electron-phonon coupling. *D*(*q*, *ω*) is the phonon Green function, with the bare phonon Green function ${D}_{0}(q,\omega )=\frac{1}{\omega -{\omega }_{p}(q)+i\eta }-\frac{1}{\omega+{\omega }_{p}(q)+i\eta }$, *ω*_*p*_ the phonon dispersion, and *η* a positive infinitesimal. When *ω* = *ω*_*p*_, it is the phonon emission, while *ω* = − *ω*_*p*_ corresponds to the phonon absorption.

For electron-magnon interaction in an itinerant ferromagnet, the spin-resolved electron self-energy in random phase approximation^69–71,119,120^ is

8 $${\Sigma }_{em}^{\sigma }(\varepsilon )={\sum }_{k,q}\int \,\frac{d\omega }{2\pi }| {m}_{k,q}{| }^{2}{G}^{\bar{\sigma }}(k-q,\varepsilon -\omega ){\chi }^{\sigma,\bar{\sigma }}(q,\omega ),$$

with *m*_*k*,*q*_ the element of the coupling matrix for electron-magnon coupling. ${\chi }^{\sigma \bar{\sigma }}(q,\omega )=\frac{{\chi }_{0}^{\sigma \bar{\sigma }}(q,\omega )}{1-{I}_{eff}{\chi }_{0}^{\sigma \bar{\sigma }}(q,\omega )}$ is the spin susceptibility, with ${\chi }_{0}^{\sigma \bar{\sigma }}(q,\omega )={\sum }_{k}\frac{f({\varepsilon }_{k\sigma })-f({\varepsilon }_{(k+q)\bar{\sigma }})}{\omega+{\varepsilon }_{k\sigma }-{\varepsilon }_{(k+q)\bar{\sigma }}+i\eta }$ the Lindhard function^121^ and *I*_*eff*_ the effective Stoner parameter. In the energy near the magnon dispersion *ω*_*m*_(*q*), ${\chi }^{\sigma \bar{\sigma }}(q,\omega )\approx \frac{{\chi }_{0}^{\sigma \bar{\sigma }}(q,\omega )}{{I}_{eff}(\partial \Re {\chi }_{0}^{\sigma \bar{\sigma }}(q,\omega )/\partial \omega {| }_{{\omega }_{m}}(\omega -{\omega }_{m}(q))+i\Im ({\chi }_{0}^{\sigma \bar{\sigma }}(q,\omega )))}- \frac{{\chi }_{0}^{\sigma \bar{\sigma }}(q,\omega )}{{I}_{eff}(\partial \Re {\chi }_{0}^{\sigma \bar{\sigma }}(q,\omega )/\partial \omega {| }_{{\omega }_{m}}(\omega+{\omega }_{m}(q))+i\Im ({\chi }_{0}^{\sigma \bar{\sigma }}(q,\omega )))}$. To make physics clear, we can define the renormalized Green function of the magnon $D(q,\omega )=\frac{{Z}^{\sigma,\bar{\sigma }}(q,\omega )}{\omega -{\omega }_{m}(q)+i\Gamma (q,\omega )}-\frac{{Z}^{\sigma,\bar{\sigma }}(q,\omega )}{\omega+{\omega }_{m}(q)+i\Gamma (q,\omega )}$, where ${Z}^{\sigma,\bar{\sigma }}(q,\omega )= \frac{{\chi }_{0}^{\sigma \bar{\sigma }}(q,\omega )}{{I}_{eff}\partial \Re {\chi }_{0}^{\sigma \bar{\sigma }}(q,\omega )/\partial \omega {| }_{{\omega }_{m}}}$ is the renormalization factor and $\Gamma (q,\omega )= \Im {\chi }_{0}^{\sigma \bar{\sigma }}(q,\omega )/(\partial \Re {\chi }_{0}^{\sigma \bar{\sigma }}(q,\omega )/\partial \omega {| }_{{\omega }_{m}})$ is the inverse lifetime of the magnon.

The Green functions of a phonon and a magnon in an itinerant ferromagnet are very different. Phonon creation or annihilation depends on the electron-phonon coupling strength. However, in addition to the electron-magnon coupling strength, magnon creation or annihilation are also weighted by the renormalization factor ${Z}^{\sigma,\bar{\sigma }}(q,\omega )$ determined by the spin-flip scattering between the spin-up and spin-down electronic structure. Generally, ${Z}^{\sigma,\bar{\sigma }}(0,\omega )\approx 0$, which means that excitation of magnons with very small q is forbidden. In the Stoner continuum, *Γ*(*q*, *ω*) is large, indicating the fast damping of the magnon. Only in the Stoner gap, *Γ*(*q*, *ω*) is very small and a long lifetime magnon exists. Moreover, ${Z}^{\sigma,\bar{\sigma }}(q,\omega )\ne {Z}^{\sigma,\bar{\sigma }}(q,-\omega )$. This inequality, together with spin-polarized electron DOS (i.e., *N*^*↑*^(*ε*) ≠ *N*^*↓*^(*ε*)), breaks the symmetry of magnon creation and annihilation compared to that of phonon.

#### Effective hamiltonian

To calculate the electron self-energy, we derived an effective electron-magnon-polaron interaction model. First of all, we start with the Hamiltonian for an itinerant ferromagnet with strong magnon-phonon coupling. This approximation is reasonable for FGT. Since it has a moderate electron-phonon coupling constant^91,122^, and a comparable electron-magnon coupling constant^91^, while the magnetoelastic coupling is strong^18^. With this approximation, we can first diagonalize the Hamiltonian for the magnon and phonon with only magnon-phonon coupling^123–127^. Then, the electron interaction with the magnon polarons was taken into account, and the electron-magnon-polaron coupling matrix elements are obtained. In the end, we obtain an effective Hamiltonian with electron-magnon-polaron interaction.

The Hamiltonian of FGT has electrons, phonons, magnons, and interactions with each other.

9 $${H}_{sample}=	 {\sum }_{\sigma,k}{\epsilon }_{k\sigma }{c}_{k\sigma }^{{{\dagger}} }{c}_{k\sigma }+{\sum }_{q}\hslash {\omega }_{p}{a}_{q}^{{{\dagger}} }{a}_{q}+{\sum }_{q}\hslash {\omega }_{m}{b}_{q}^{{{\dagger}} }{b}_{q}\\ 	+{\sum }_{\sigma,k,q}{g}_{k,q}{c}_{(k+q)\sigma }^{{{\dagger}} }{c}_{k\sigma }({a}_{q}+{a}_{-q}^{{{\dagger}} })\\ 	+{\sum }_{k,q}{m}_{k,q}({c}_{(k+q)\uparrow }^{{{\dagger}} }{c}_{k\downarrow }{b}_{q}+{c}_{(k+q)\downarrow }^{{{\dagger}} }{c}_{k\uparrow }{b}_{-q}^{{{\dagger}} })\\ 	+{\sum }_{q}{\lambda }_{q}({a}_{q}+{a}_{-q}^{{{\dagger}} })({b}_{q}+{b}_{-q}^{{{\dagger}} }).$$

with *g*_*k*,*q*_ the electron-phonon coupling matrix element, *m*_*k*,*q*_ the electron-magnon coupling matrix element, and *λ*_*q*_ the magnon-phonon coupling strength or magnetoelastic coupling. Note that the magnon-phonon coupling term can be diagonalized by Bogoliubov transformation^123,124^. In fact, $({a}_{q}{b}_{-q}^{{{\dagger}} }+{a}_{-q}^{{{\dagger}} }{b}_{q})$ conserves the total number of bosons, while $({a}_{-q}^{{{\dagger}} }{b}_{-q}^{{{\dagger}} }+{a}_{q}{b}_{q})$ does not, which is usually ignored^125–127^.

The electron-phonon coupling matrix element *g*_*k*,*q*_ is usually small, the electron-magnon coupling matrix element *m*_*k*,*q*_ is large and of the order of the exchange interaction between the local spin *S* and the electron spin *σ* (${J}_{{{{\rm{ex}}}}}\vec{\sigma }\vec{S}$)^69^, but is forbidden for low-energy and momentum transfer due to the Stoner gap, that is, there are no pairs of states with small energy difference in bands of opposite spin. The hybridized magnon-phonon mixes spin degrees of freedom with lattice vibrations such that the spin in the lattice becomes ill defined. We will have terms containing magnon-phonon mixing in the Hamiltonian, mainly at the crossings of magnons and phonons.

Firstly, without electron-magnon and electron-phonon interactions, the magnon-phonon interactions with a total number conservation term only can be diagonalized to magnon polarons, following refs. ^125–127^, i.e., *A*_*q*_ = *u*_*q*_*a*_*q*_ + *v*_*q*_*b*_*q*_, *B*_*q*_ = *u*_*q*_*b*_*q*_ − *v*_*q*_*a*_*q*_, where ${u}_{q}=\sqrt{\frac{{\omega }_{s}+{\omega }_{\delta }}{2{\omega }_{s}}}$ and ${v}_{q}=\sqrt{\frac{{\omega }_{s}-{\omega }_{\delta }}{2{\omega }_{s}}}$ with ${\omega }_{s}=\sqrt{{\omega }_{\delta }^{2}+4{\lambda }_{q}^{2}}$ and *ω*_*δ*_ = (*ω*_*m*_ − *ω*_*p*_) for *λ*_*q*_ ≠ 0 ^125,126^. When *λ*_*q*_ = 0, *u*_*q*_ = 1 and *v*_*q*_ = 0. Note that ${u}_{q}^{2}+{v}_{q}^{2}=1$ with *u*_*q*_ and *v*_*q*_ ∈ [0, 1], indicating the total number conservation of magnon and phonon. At the avoided level crossing, the bosons are equally weighted as ${A}_{q}=\frac{{a}_{q}+{b}_{q}}{\sqrt{2}}$ and ${B}_{q}=\frac{{b}_{q}-{a}_{q}}{\sqrt{2}}$ with $u=v=\sqrt{1/2}$.

Secondly, taking the electron-magnon and electron-phonon interactions into account by using the magnon-polaron basis, we can obtain the Hamiltonian with the electron-magnon-polaron interaction as

10 $$H=	 {\sum }_{\sigma,k}{\epsilon }_{k\sigma }{c}_{k\sigma }^{{{\dagger}} }{c}_{k\sigma }+{\sum }_{q}\hslash {\omega }_{q}^{A}{A}_{q}^{{{\dagger}} }{A}_{q}++ {\sum }_{q}\hslash {\omega }_{q}^{B}{B}_{q}^{{{\dagger}} }{B}_{q} \\ 	+{\sum }_{k\sigma,q{\sigma }^{{\prime} }}({\alpha }_{k,q}^{{\sigma }^{{\prime} }\sigma }({A}_{q}+{A}_{-q}^{{{\dagger}} })+{\beta }_{k,q}^{{\sigma }^{{\prime} }\sigma }({B}_{q}+{B}_{-q}^{{{\dagger}} })){c}_{(k+q){\sigma }^{{\prime} }}^{{{\dagger}} }{c}_{k\sigma },$$

where electron-magnon-polaron coupling matrix in spin space is below

11 $$\begin{array}{l}\,{\alpha }_{k,q}=\left(\begin{array}{cc}{g}_{k,q}{u}_{q} & {m}_{k,q}{v}_{q}\\ {m}_{k,q}{v}_{q} & {g}_{k,q}{u}_{q}\end{array}\right),\\ {\beta }_{k,q}=\left(\begin{array}{cc}-{g}_{k,q}{v}_{q} & {m}_{k,q}{u}_{q}\\ {m}_{k,q}{u}_{q} & -{g}_{k,q}{v}_{q}\end{array}\right).\end{array}$$

The above Hamiltonian describes the electron-magnon-polaron interactions. It is similar to electron-phonon or electron-magnon interactions. Note, however, that the matrix elements are now mixed, i.e., inelastic excitations are possible with and without spin flip. The strict selection rules that forbid magnon excitation in the Stoner gap are lifted. Therefore, ISTS can be used to efficiently detect magnon polarons.

Based on the above effective Hamiltonian, we can derive the electron self-energy as

12 $$\Sigma (\varepsilon )={\sum }_{k\sigma,q{\sigma }^{{\prime} }}^{\nu }\int \,\frac{d\omega }{2\pi }| {\gamma }_{\nu,k,q}^{\sigma {\sigma }^{{\prime} }}{| }^{2}{G}^{{\sigma }^{{\prime} }}(k-q,\varepsilon -\omega )D(q,\omega ).$$

Here, *ν* = *A*, *B* is the magnon-polaron band index, and *D*(*q*, *ω*) is now the Green function of the magnon-polaron. When ${\sigma }^{{\prime} }=\sigma$, $D(q,\omega )=\frac{1}{\omega -{\omega }_{mp}(q)+i{\Gamma }_{ep}}-\frac{1}{\omega+{\omega }_{mp}(q)+i{\Gamma }_{ep}}$, where *ω*_*mp*_(*q*) is the magnon polaron dispersion and *Γ*_*ep*_ the inverse lifetime due to the electron-phonon interaction. However, when ${\sigma }^{{\prime} }=\bar{\sigma }$, $D(q,\omega )=\frac{Z(q,\omega )}{\omega -{\omega }_{mp}(q)+i{\Gamma }_{em}}-\frac{Z(q,\omega )}{\omega+{\omega }_{mp}(q)+i{\Gamma }_{em}}$ with *Γ*_*em*_ the inverse lifetime due to the electron-magnon interaction.

#### ISTS for magnon polaron excitations

Here we start from the effective Hamiltonian obtained to calculate the derivative of the imaginary part of electron self-energy as a result of electron-magnon polaron interactions, which incorporate electrons, phonons, magnons and interactions between each other. As an implementation with ab initio dispersions of all these three quasiparticles is currently not feasible due to computational limits, we focus on the physics of this problem using empirical models for these quasiparticles, i.e., the free electron model with exchange splitting, the Debye model for acoustic phonon, and the quadratic dispersion for magnon. To highlight the magnon-phonon coupling induced selection, we use minimal assumptions for the interactions between electron, phonon and magnon, i.e., treating the electron-phonon coupling matrix element *g*_*k*,*q*_ (≈ 0.01 eV for a moderate electron-phonon interaction^91^), the electron-magnon coupling matrix element *m*_*k*,*q*_ ($\approx \frac{{\Delta }_{ex}}{S}=1.4$ eV with *Δ*_*ex*_ = 1.05 eV and *S* = 0.74 for FGT^18^) and the magnon-phonon coupling matrix element *λ*_*q*_ as constants. Note that no magnon-phonon coupling strength is reported for FGT, but for FePS_3,_ the reported hybridization gap is  ≈ 0.25 meV^66^. In the Stoner gap, ${Z}^{\sigma,\bar{\sigma }}(q,\omega )(\approx 0.001) < < 1$ and *Γ*(*q*, *ω*) is very small. Then, only the electron-magnon-polaron coupling matrix is *q*-dependent, i.e., *α*_*q*_ and *β*_*q*_. Moreover, this momentum dependence originates from the *q*-dependent *u*_*q*_ and *v*_*q*_, which depend on the magnon-phonon coupling. With these approximations, we obtained the derivative of the imaginary part of the self-energy as below

13 $$-\partial \Im \Sigma (\varepsilon )/\partial \varepsilon \approx {{{\rm{sgn}}}}(\varepsilon ){\sum }_{\nu,\sigma }[| {\gamma }_{\nu }^{\sigma,\sigma }{| }^{2}{N}^{\sigma }({E}_{F})+| {\gamma }_{\nu }^{\sigma,\bar{\sigma }}{| }^{2}| {Z}^{\sigma,\bar{\sigma }}| {N}^{\bar{\sigma }}({E}_{F})]{F}_{\nu }(\varepsilon ).$$

Here, *F*_*ν*_(*ε*) is the magnon-polaron DOS with band index, and *N*^*σ*^(*E*_*F*_) is the spin-resolved electron DOS at the Fermi energy. According to Eq. (6), *d*^2^*I*/*dU*^2^ ∝ Eq. (13). We note that the damping of the phonon and magnon due to electrons is not considered, resulting in an energy-dependent decay behavior as observed in experiments.

## Supplementary information

Supplementary Information

Transparent Peer Review file

## Data Availability

The data supporting the findings of this study are provided in the Article and Supplementary Information. The raw data generated in this study have been deposited in Radar4KIT under DOI: 10.350970/atenzyzbf6qsyyy.
