# Supplementary material for: Observation of magnon polarons in the van der Waals itinerant ferromagnet Fe3GeTe2
Source: Nat Commun. 2026 Jun 20;17:5461. doi: 10.1038/s41467-026-74465-3 (PMC13283220; doi:10.1038/s41467-026-74465-3)
Supplement: Supplementary file 1 — Supplementary Information [file 41467_2026_74465_MOESM1_ESM.pdf]

Supplementary Information
Observation of magnon polarons in van-der-Waals itinerant ferromagnet Fe_3GeTe_2

Qili Li[1,*,†], Namrata Bansal[1,*], Paul Nufer[1], Lichuan Zhang[2,3], Amir-Abbas Haghighirad[4], Christoph Sürgers[1], Yuriy Mokrousov[3,5], and Wulf Wulfhekel[1,4]

[1]Physikalisches Institut, Karlsruhe Institute of Technology, Karlsruhe, Germany
[2]School of Physics and Electronic Engineering, Jiangsu University, Zhenjiang, China
[3]Peter Grünberg Institut (PGI-1) and Institute for Advanced Simulation (IAS-1) Forschungszentrum Jülich GmbH, Jülich, Germany
[4]Institute for Quantum Materials and Technologies, Karlsruhe Institute of Technology, Karlsruhe, Germany
[5]Institute of Physics, Johannes Gutenberg-University Mainz, Mainz, Germany
[*]These authors contributed equally to this work.
[†]Corresponding author: qili.li@kit.edu

Contents

1 Magnetic-field dependence of quasiparticle excitations 2

2 Temperature dependence of quasiparticle excitations 6

3 Convolution of ISTS 7

4 Identify magnon-phonon band crossings 7

5 Model calculations for optical phonon with and without magnon-phonon coupling 8

6 Temperature dependence of the in-plane electrical resistivity of the Fe_3GeTe_2 single crystal 9

1 Magnetic-field dependence of quasiparticle excitations

Here we present the magnetic-field dependence of excitations under an out-of-plane magnetic field up to 5 T measured with W tip at 45 mK. Experimentally, the energy peak at 0.8 meV shifts upward to 1.1 meV at 3 T and 1.25 meV at 5 T (see Supplementary Fig. 1), in quantitative agreement with the expected Zeeman energy shifts of 0.35 meV (3 T) and 0.58 meV (5 T). This agreement confirms that the low-energy mode follows the expected magnetic scaling and provides a direct calibration of the magnonic energy scale in our measurements. By contrast, the higher-energy peaks shift downward with increasing magnetic field (see Supplementary Fig. 1). Such opposite field dependence cannot be explained within a bare magnon picture, in which all magnon modes shift monotonically upward with field. Note that the 0.3 mV peak does not change with magnetic field, which originates from the dynamical Coulomb blockade [1–4].

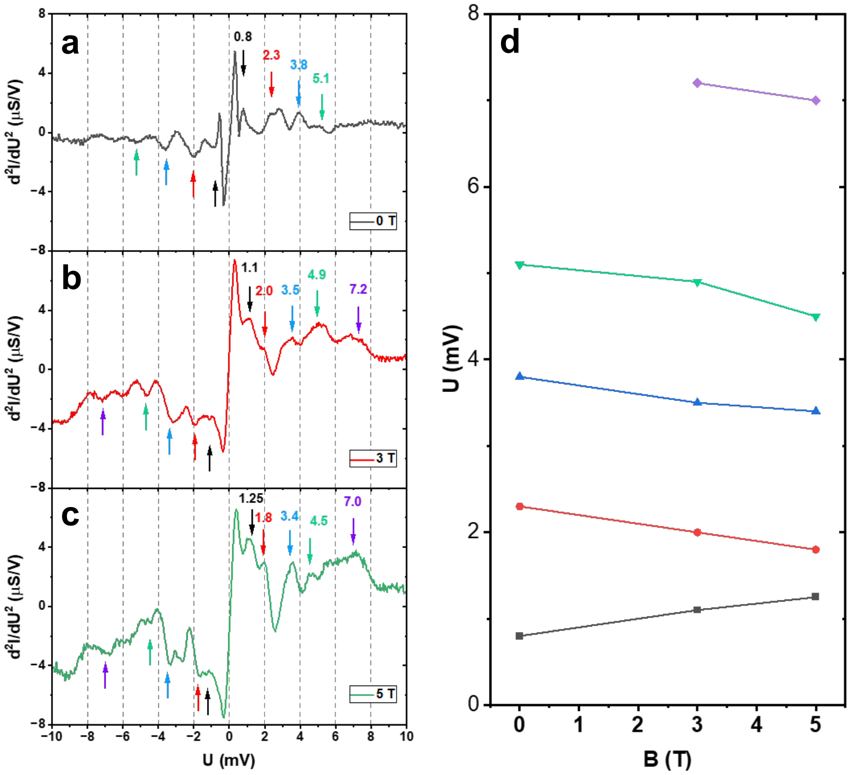

Supplementary Figure 1: Magnetic-field dependence. d^2I/dU^2 measured at 45 mK under different magnetic fields at 0 T (black curve in **a**), 3 T (red curve in **b**), and 5 T (green curve in **c**). The shifts of peaks are plotted in **d**. The tunneling conditions: 0 T (U = 20 mV, I = 2 nA with U_{mod}=0.5 mV), 3 T (U = 10 mV, I = 2 nA with U_{mod}=0.5 mV), 5 T (U = 10 mV, I = 2 nA with U_{mod}=0.5 mV). The modulation frequency was 3.421 kHz and was the same for the different magnetic fields.

To illustrate the magnetic-field dependence of these excitations, we further showcase the model calculations for magnon-phonon hybridization. Generally, the magnetic field shifts the magnon dispersion up because of the Zeeman energy, whereas the phonon dispersion is independent of the magnetic field. According to the magnon–phonon hybridization scenario, different hybrid branches exhibit distinct magnetic-field responses (see Supplementary Figs. 2-4). For magnon-acoustic phonon coupling, there are two avoided band crossings. When the energy of these two avoided band

crossings are far apart from each other, the increasing magnetic field lifts the magnon dispersion making the energy of lower crossing increase while the energy of the higher crossing decreases (see Supplementary Figure 2). However, when the energy of these two avoided band crossings are close with each other, the increasing magnetic field lifts the magnon dispersion leading to the emergence and broadening of the two magnon-polaron peaks. Eventually, no crossing and no magnon polaron exists anymore (see Supplementary Figure 3). For magnon-optical phonon coupling, there is only one avoided band crossing. The increasing magnetic field lifts the magnon dispersion, whereas the energy of magnon polaron decreases (see Supplementary Figure 4).

Our model calculations for magnon-phonon hybridization scenario demonstrate that the first avoided crossing with acoustic phonons shifts to higher energy with increasing magnetic field, whereas the second avoided crossing with acoustic phonons and the avoided crossing with optical phonons shifting to lower energy. This can well explain both the upward shift for the lowest-energy peak at 0.8 meV and the downward shifts of high-energy peaks in Supplementary Fig. 1d.

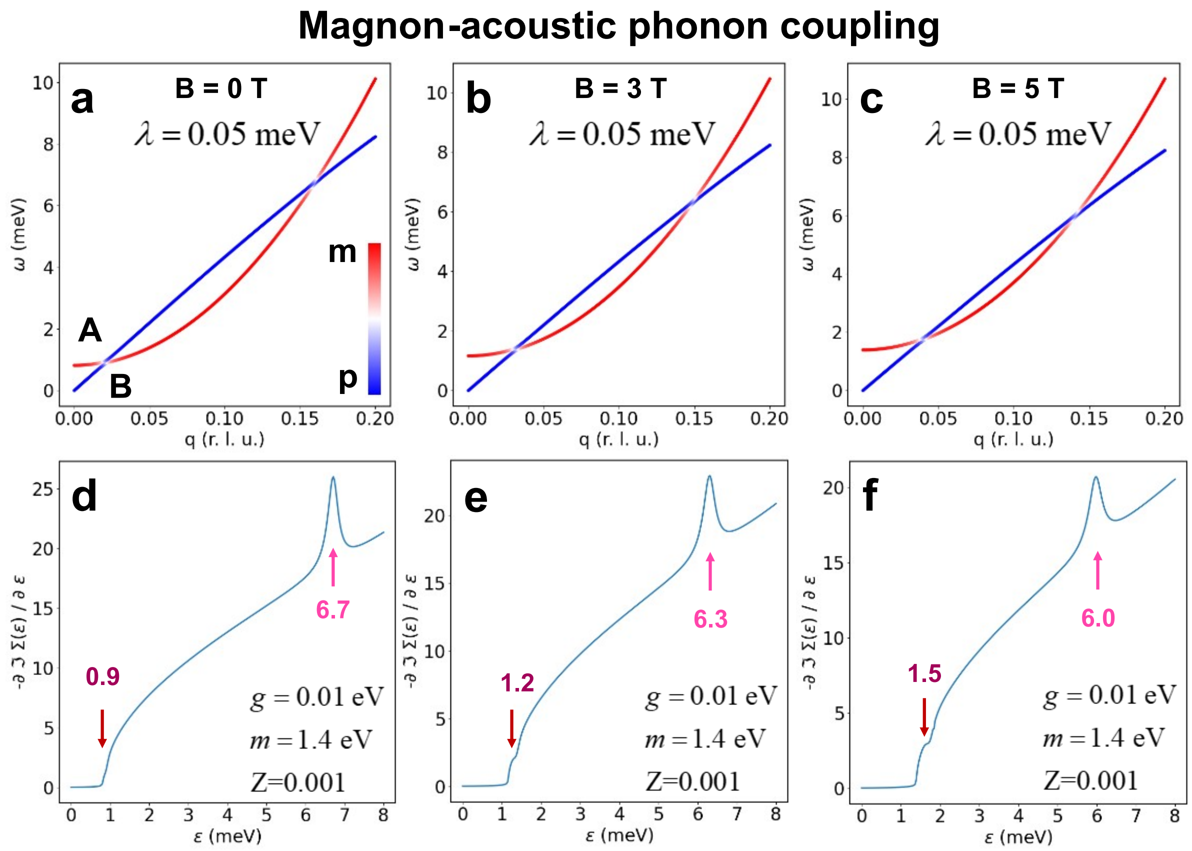

Supplementary Figure 2: Magnon-acoustic phonon coupling under an applied magnetic field. For magnon-acoustic phonon coupling, there are two avoided band crossings. When the energies of these two avoided band crossings are far away from each other, the increasing magnetic field lifts the magnon dispersion making the energy of lower crossing increases while the energy of the higher crossing decrease.

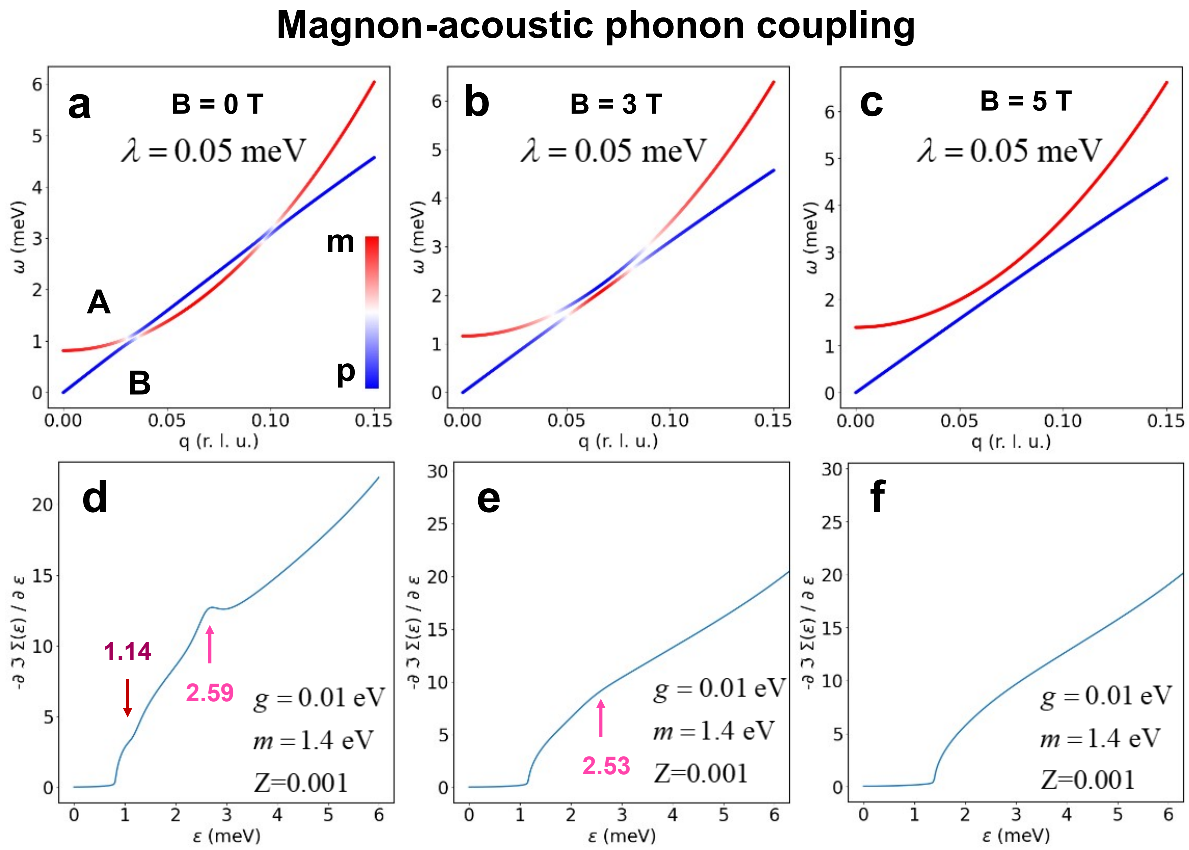

Supplementary Figure 3: Magnon-acoustic phonon coupling under an applied magnetic field. For magnon-acoustic phonon coupling, there are two avoided band crossings. When the energies of these two avoided band crossings are close to each other, the increasing magnetic field lifts the magnon dispersion driving the emergence and broadening of the two magnon polaron peaks. Eventually, no crossing is observed and a magnon polaron is not created.

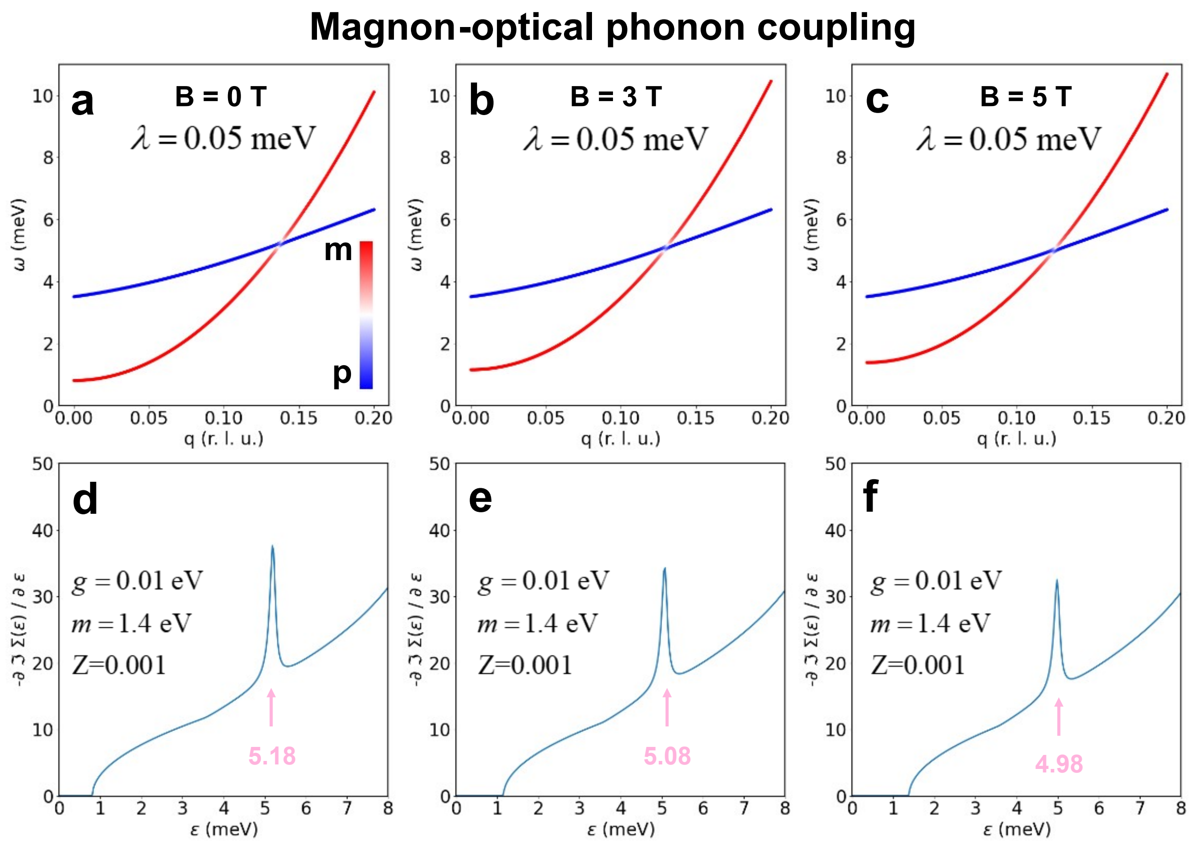

Supplementary Figure 4: Magnon-optical phonon coupling under an applied magnetic field. For magnon-optical phonon coupling, there is only one avoided band crossing. The increasing magnetic field lifts the magnon dispersion somewhat, whereas the energy of magnon polaron decreases.

2 Temperature dependence of quasiparticle excitations

Supplementary Figure 5 shows low-energy excitations at 775 mK with W tip. Three peak-dip pairs can still be seen i.e., at 0.8, 2.0, and 3.6 mV with an experimental resolution of ±0.63 meV (see arrows in Supplementary Fig. 5), which are consistent with the measurements at 40 mK shown in Fig. 2c. However, the intensities of these peaks change significantly. We can expect that at even higher temperatures all individual peaks will merge as one broad peak.

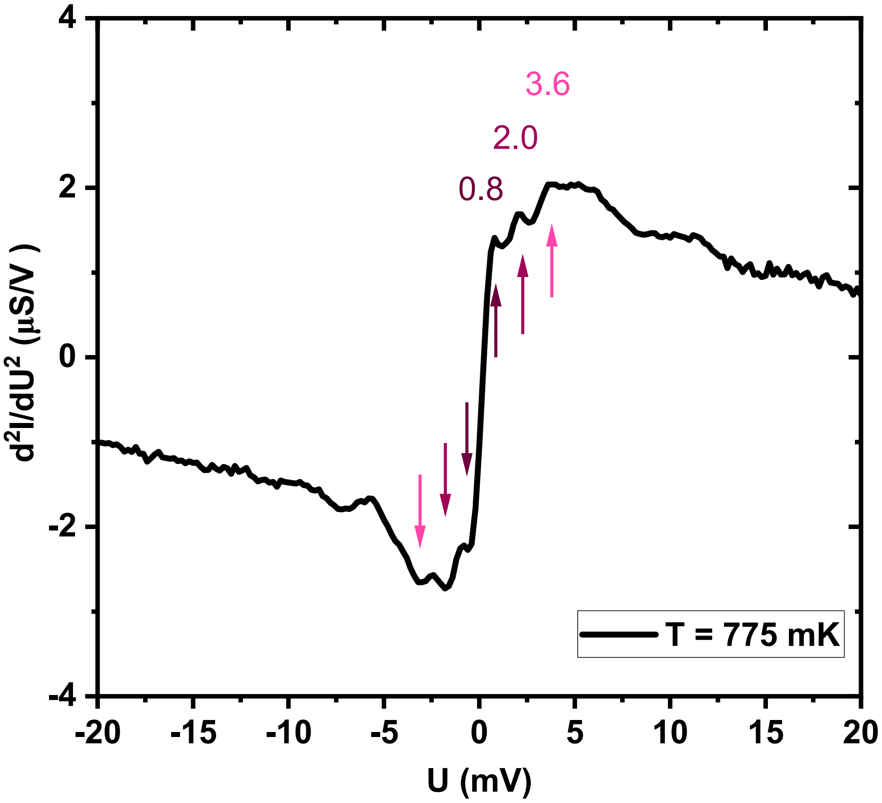

Supplementary Figure 5: Temperature dependence. $\mathrm{d}^2I/\mathrm{d}U^2$ spectra measured at 775 mK. The tunneling condition was $U = 20$ mV, $I = 3$ nA, and U_{mod}=1 mV at 6.551 kHz.

3 Convolution of ISTS

For spin-polarized (SP) ISTS measurements, we used a larger modulation than that of ISTS to obtain the inelastic signal. To compare the SP-ISTS with ISTS, we follow the work of Klein et al. [5] to calculate the convolution of ISTS (Fig. 2c). According to ref. [5], the lock-in amplifier measured $(\frac{d^2I}{dU^2})_{lock-in} = (\frac{d^2I}{dU^2})_{exact} * \phi(E)$, with the instrumental function $\phi = \frac{8}{3\pi}\frac{1}{(eU_{mod})^4}(e^2U_{mod}^2 - E^2)^{3/2}$ for $|E| <= eU_{mod}$, and $\phi(E) = 0$ for $|E| > eU_{mod}$. Note that the thermal broadening is negligible since both SP-ISTS and ISTS were measured at 40 mK.

For simplicity, the modulation results in an experimental resolution $\pm 0.6U_{mod}$ [5]. Therefore, the ISTS in Fig. 2c with modulation amplitude $U_{mod} = 0.5$ mV has an energy resolution ± 0.3 meV, whereas the SP-ISTS in Fig. 3(d,e) has an energy resolution ± 0.6 meV.

4 Identify magnon-phonon band crossings

With the dispersions of the phonon and the magnon, we calculated their band crossings as follows. We defined an intensity function to characterize the band crossings. $I(\omega_m(q)) = \sum_\nu \frac{\eta}{(\omega_m(q)-\omega_p^\nu(q))^2+\eta^2}$, with a small broadening parameter $\eta = 0.05$ meV. ν is the phonon band index. A peak appears in $I(\omega_m(q))$ when the phonon band crosses with the magnon band $(\omega_p(q) = \omega_m(q))$.

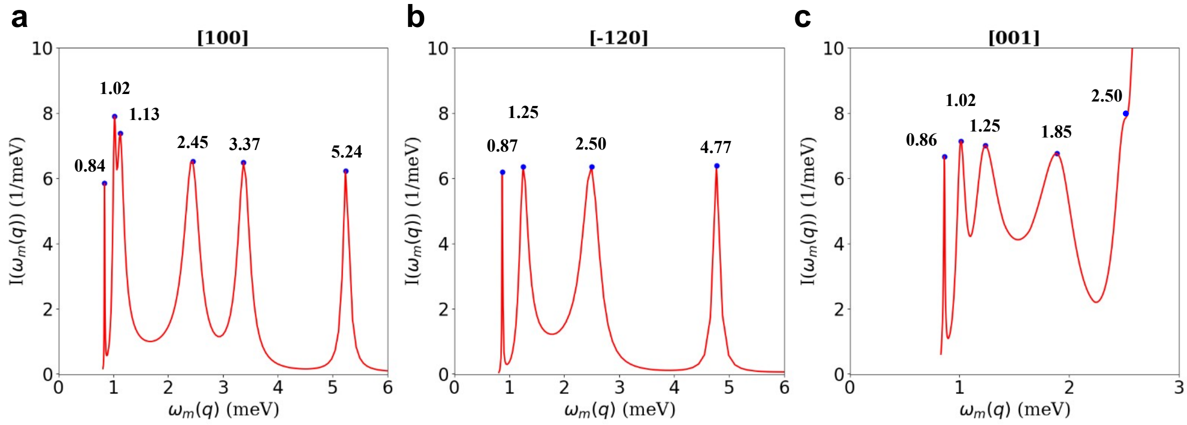

Supplementary Figure 6: Identify magnon-phonon band crossings. **a-c** are band crossings in [100], [-120] and [001] directions, respectively.

5 Model calculations for optical phonon with and without magnon-phonon coupling

Here we demonstrate that the lowest optical phonon band crosses with the magnon band, resulting in the magnon polaron excitation at 5.18 meV which agrees with our experimental results. The parameters used for the model calculations are the same as in Figure 5 in the main text. Generally, the optical phonon has one crossing with magnon band. In the case of magnon-phonon coupling, the avoided crossing results in a peak in ISTS. Note that the DOS does not change with magnon-phonon coupling (see Figure 5(b,e)).

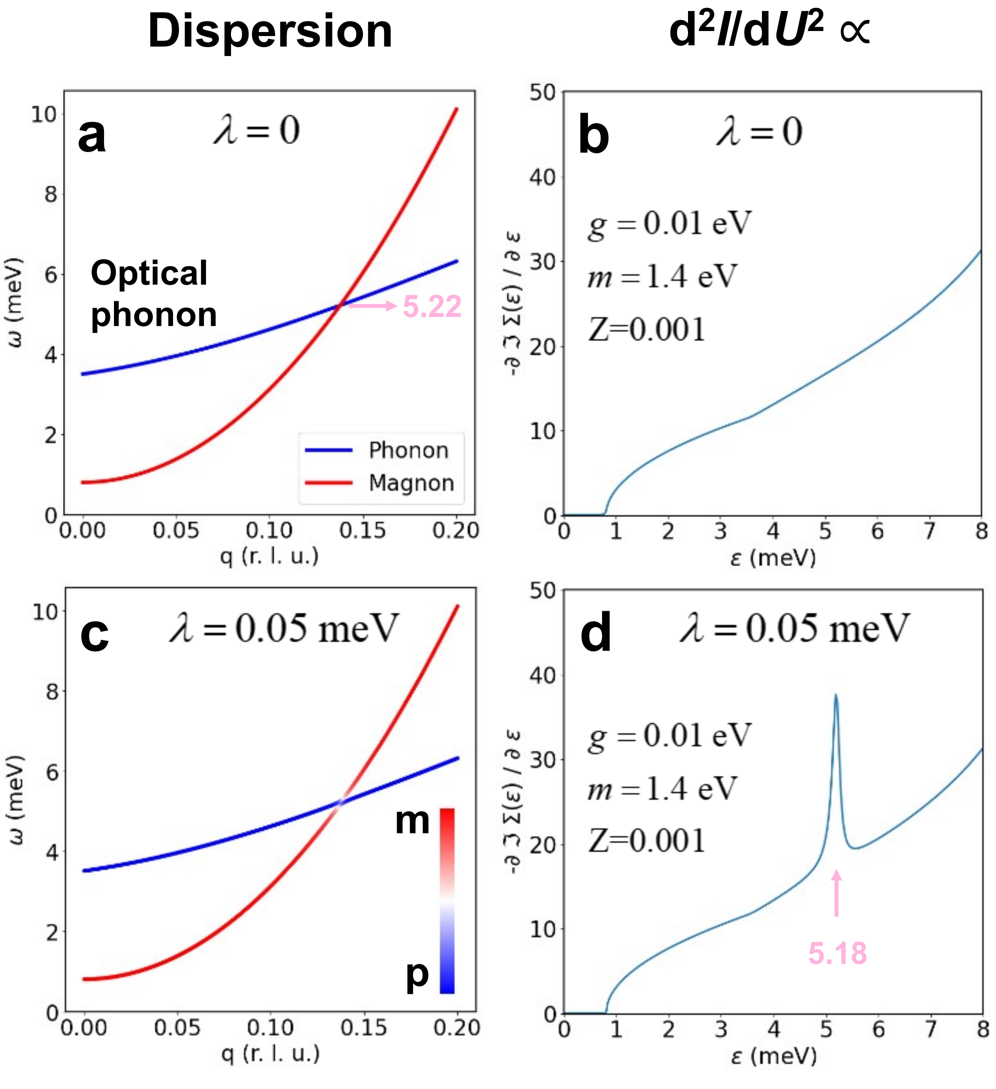

Supplementary Figure 7: Model calculations for optical phonon without and with magnon-phonon coupling. **a** Band crossing for magnon and optical phonon. **b** The derivative of electron self-energy without magnon-phonon coupling. **c** Avoided band crossing between magnon and optical phonon. **d** The derivative of electron self-energy i.e., ISTS for magnon polaron.

6 Temperature dependence of the in-plane electrical resistivity of the Fe_3GeTe_2 single crystal

We measured the temperature dependence of the in-plane electrical resistivity of the Fe_3GeTe_2 single crystal. Supplementary Figure 8 displays the resistivity measured after magnetically saturating the FGT sample followed by warming up, displaying a sizable increase in resistivity above about 10 K and a kink at the Curie temperature $T_C = 205$ K. Notably, there is no clear maximum of the resistivity is observed that would indicate a Kondo lattice behavior at temperatures above 10 K [6, 7].

In addition, the inset shows a $-\log(T)$ behavior at temperatures below 10 K. For this to be due to a Kondo effect, the magnetoresistance should be negative and the log slope should decrease with increasing magnetic field. However, what we see is first a positive magnetoresistance at 3 T, and then a decrease at 6 T and 9 T excluding a low-temperature Kondo effect. Instead, we suggest that this behavior comes from electron-electron interaction [8] or electron-magnon-polaron interaction.

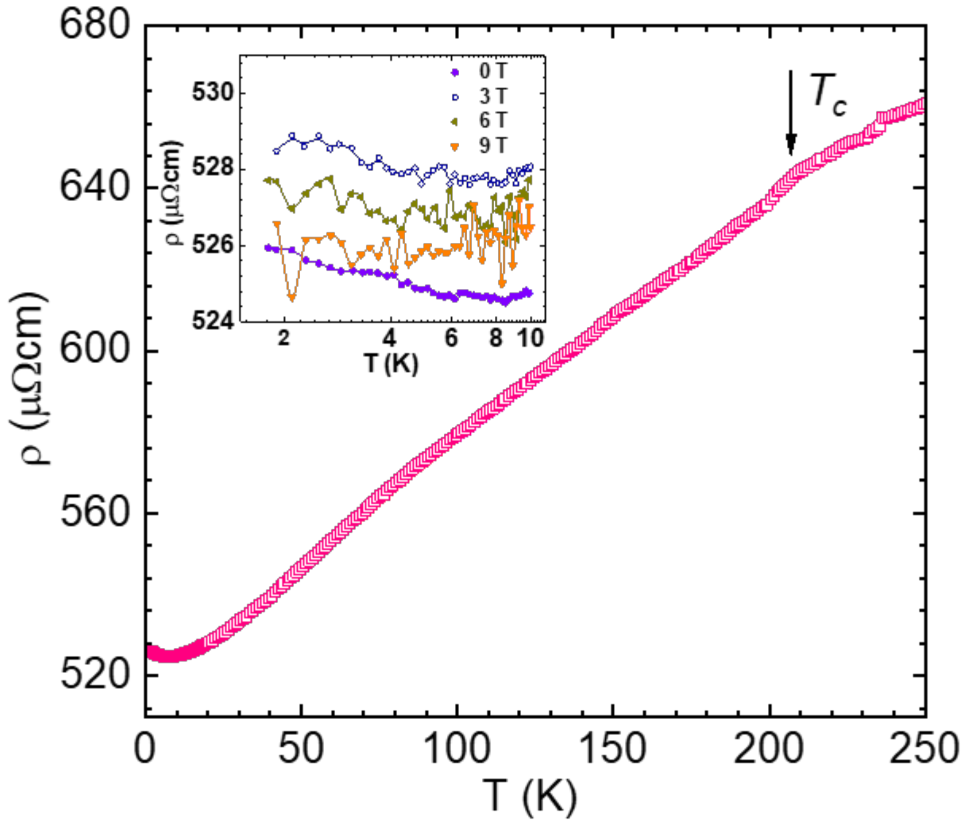

Supplementary Figure 8: Temperature dependence of the in-plane electrical resistivity of the Fe_3GeTe_2 single crystal. Resistivity vs temperature after magnetically saturating the FGT sample followed by warming up. Inset shows the semi-logarithmic plot of the resistivity at low temperatures under different magnetic fields.

References

[1] Schackert, M. *et al.* Local measurement of the eliashberg function of Pb islands: Enhancement of electron-phonon coupling by quantum well states. *Phys. Rev. Lett.* **114**, 047002 (2015). URL https://journals.aps.org/prl/abstract/10.1103/PhysRevLett.114.047002.

[2] Ast, C. R. *et al.* Sensing the quantum limit in scanning tunnelling spectroscopy. *Nat. Commun.* **7**, 13009 (2016). URL https://doi.org/10.1038/ncomms13009.

[3] Senkpiel, J. *et al.* Dynamical coulomb blockade as a local probe for quantum transport. *Phys. Rev. Lett.* **124**, 156803 (2020). URL https://www.ncbi.nlm.nih.gov/pubmed/32357030.

[4] Esat, T., Ternes, M., Temirov, R. & Tautz, F. S. Electron spin secluded inside a bottom-up assembled standing metal-molecule nanostructure. *Phys. Rev. Res.* **5**, 033200 (2023). URL https://link.aps.org/doi/10.1103/PhysRevResearch.5.033200.

[5] Klein, J., Léger, A., Belin, M., Défourneau, D. & Sangster, M. J. L. Inelastic-electron-tunneling spectroscopy of metal-insulator-metal junctions. *Phys. Rev. B* **7**, 2336–2348 (1973). URL https://journals.aps.org/prb/abstract/10.1103/PhysRevB.7.2336.

[6] Lavagna, M., Lacroix, C. & Cyrot, M. Electrical resistivity of the kondo lattice. *Journal of Applied Physics* **53**, 2055–2057 (1982). URL https://doi.org/10.1063/1.330742.

[7] Zhang, Y. *et al.* Emergence of kondo lattice behavior in a van der Waals itinerant ferromagnet, Fe_3GeTe_2. *Sci. Adv.* **4**, eaao6791 (2018). URL https://www.science.org/doi/full/10.1126/sciadv.aao6791.

[8] Gopalakrishnan, B. *et al.* Electronic transport in magnetically ordered $Mn_5Si_3C_x$ films. *Phys. Rev. B* **77**, 104414 (2008). URL https://link.aps.org/doi/10.1103/PhysRevB.77.104414.
