# Supplementary material for: Observation of magnon polarons in the van der Waals itinerant ferromagnet Fe3GeTe2
Source: Nat Commun. 2026 Jun 20;17:5461. doi: 10.1038/s41467-026-74465-3 (PMC13283220; doi:10.1038/s41467-026-74465-3)
Supplement: Supplementary file 2 — Transparent Peer Review file [file 41467_2026_74465_MOESM2_ESM.pdf]

nature portfolio

Peer Review File

Observation of magnon polarons in van-der-Waals itinerant ferromagnet Fe_3GeTe_2

Corresponding Author: Dr Qili Li

This file contains all reviewer reports in order by version, followed by all author rebuttals in order by version.

Version 0:

Reviewer comments:

Reviewer #1

(Remarks to the Author)
In this manuscript, the authors report low-temperature spin-polarized and non–spin-polarized scanning tunnelling spectroscopy (STS) measurements on Fe_3GeTe_2 (FGT), revealing several discrete inelastic features that they interpret as signatures of magnon–phonon hybridization (magnon polarons). By comparing the observed energies with calculated magnon and phonon dispersions, the authors argue that the peaks correspond to hybrid modes emerging at avoided crossings between acoustic magnons and phonons. Additional magnetic-field and domain-wall–dependent measurements are presented to support the magnonic component of the excitations, and a simplified model for the local density of states is constructed. The topic is timely and the experimental technique is sophisticated. If firmly validated, this work would represent a significant advance. However, at present, several key aspects of the central interpretation remain insufficiently substantiated.
Major Comments
1. The identification of magnon–phonon hybridization is based mainly on energy coincidence with calculated crossings and would benefit from more quantitative support distinguishing hybrid modes from simple magnon–phonon overlap.
2. The magnetic-field evolution of the inelastic features is presented qualitatively and requires a more systematic quantitative analysis.
3. The spin-polarized STS contrast across domain walls is modest, and the robustness and reproducibility of the spin-dependent signals need to be better documented.
4. The applicability of the adopted magnon dispersion to Fe-deficient FGT samples requires stronger justification.
5. A clearer distinction between hybrid excitations and purely phononic van Hove singularities is needed.
Minor Comments
1. The manuscript is rather long and includes extensive theoretical exposition; the authors may consider streamlining some sections for clarity.
2. Some figures would benefit from clearer labeling (e.g., consistent color coding between experiment and theory).
3. Please clarify whether the use of “spin gap,” “Stoner gap,” and “magnon gap” refers to the same quantity or to distinct physical concepts.
4. The temperature stability during long spectroscopy measurements should be stated explicitly.
5. In several places, the error bars or uncertainties of peak positions are not indicated.

Reviewer #2

(Remarks to the Author)
The manuscript presents a combination of experimental ISTS data using unpolarized andspin-polarized tips, and theoretical modeling to demonstrate magnon-phonon hybridization in Fe3GeTe2. The concept of exploring hybrid excitations in a metallic van der Waals ferromagnet is innovative and aligns with the scope of Nature Communications. However, the evidence presented does not meet the journal's high standards, primarily due to limitations in data depth, methodological rigor, and interpretive robustness. Below, I outline specific concerns that preclude acceptance in its current form.

(1) The ISTS spectra in Fig. 2(d) reveal several distinct peaks (0.3, 0.8, 2.5, 3.8, and 5.2 meV), which are interpreted as signatures of magnon-polarons. However, the data rely on a relatively small dataset (averaged over 121 curves in a 4×4

nm2 area). This raises questions about statistical significance and reproducibility. Claims of this importance require broader spatial mapping and replication across multiple samples to rule out localized artifacts.

(2) The large-scale topography in Fig. 2(a) indicates an unstable tip condition, likely due to surface contamination. Tip instability can introduce artificial features in STS measurements, potentially explaining the multiple peaks observed in ISTS. Such artifacts might also exhibit domain-wall dependence, as seen in the dI/dV maps. The authors should repeat measurements with a cleaner sample and more stable tip conditions to ensure reliability.

(3) The magnetic-field-dependent ISTS data (Supplementary Fig. 1) are limited to three field values (0T, 3T, 5T), with no clear trend established between peaks across fields. The relationship between peaks at 0 T and those under higher fields is ambiguous. Additionally, the absence of field-dependent ISTS using spin-polarized tips weakens the magnetic origin argument.

(4) The ISTS peaks obtained with W tips (Fig. 2(d)) appear inconsistent with those from spin-polarized tips (Fig. 3(b)). To validate reliability, the authors should directly compare raw ISTS spectra for both tips (for spin-polarized tip, the data on aligned and anti-aligned domains should be shown respectively) in a single figure.

(5) While Fig. 3 claims domain-wall effects in spin-polarized ISTS, the contrast changes are subtle and susceptible to tip-related artifacts. The description of peak-dip asymmetry based on color plots (Fig. 3(b)) is not visually convincing. Raw ISTS spectra should be plotted to clarify these features.

(6) The ISTS map at 10 mV (Fig. 3(c)) shows a weak but perceptible transition at the domain wall, potentially due to low signal intensity. The relevance of this transition to the magnon-polaron narrative remains unexplained, undermining its interpretive value.

(7) The paper omits verification of the spin polarization of Cr-coated tips before and after ISTS measurements.

(8) The claim that "For the Kondo effect to appear, the system needs to obey time-reversal symmetry" is incorrect; Kondo effects can coexist with time-reversal symmetry breaking, as demonstrated in systems like CeRh6Ge4 (Nature 579, 51, 2020). This erroneous premise weakens the argument against alternative interpretations of the dip-like feature in Fe3GeTe2.

(9) The model calculations for electron self-energy and magnon-phonon coupling rely on empirical parameters rather than ab initio-derived values. This simplification fails to capture momentum-dependent hybridization, reducing the quantitative accuracy of predicted band crossings and ISTS peaks.

(10) The resistivity data (Supplementary Fig. 5) show a kink near 80 K, but the connection to magnon-polaron damping is speculative without direct correlation to ISTS energies or robust theoretical support.

Despite the novel premise, the manuscript lacks the data richness, methodological rigor, and interpretive accuracy required for Nature Communications. The concerns outlined above (particularly regarding data quality and statistical validity) significantly undermine the claims of magnon-polaron observation. I recommend a substantial revision addressing these points, with potential resubmission to a more specialized journal after further validation.

Version 1:

Reviewer comments:

Reviewer #1

(Remarks to the Author)
The authors have carefully revised the manuscript and addressed several points raised in the previous round. The work presents high-quality ISTS measurements on Fe3GeTe2 and tackles a timely and interesting problem. The comparison between the observed inelastic features and calculated magnon–phonon dispersions is clearly presented, and the interpretation in terms of magnon–phonon hybridization is physically reasonable. However, after re-evaluating the revised manuscript, I feel that the central claim, the identification of magnon–phonon hybrid quasiparticles, is still supported primarily by indirect evidence. While the revision has improved the presentation, several aspects of the interpretation would benefit from further clarification to more firmly establish the proposed picture:
1. The assignment of the observed peaks to magnon–phonon hybrid modes is mainly based on their correspondence with calculated avoided crossings. While this agreement is suggestive, it remains somewhat indirect, and it would be helpful to clarify how these features can be distinguished from a superposition of magnon and phonon excitations.
2. The magnetic-field-dependent and spin-polarized measurements support a magnonic contribution. A more quantitative comparison with expected magnetic energy scales (e.g., field-induced shifts) would help to assess whether all observed modes consistently follow a magnonic origin.
3. Although possible alternative origins (e.g., phonon-related features or tip effects) are discussed, it remains unclear whether the current analysis uniquely favors the hybridization scenario over these alternatives. Further clarification would help to reinforce the proposed interpretation.
4. The reported spectra show local reproducibility; however, the extent to which these features are spatially uniform beyond the measured regions remains unclear. Additional discussion of spatial consistency would help establish the intrinsic nature

of the observed excitations.
5. The inelastic peaks exhibit noticeable differences in intensity, including a relatively strong low-energy feature. Further discussion on whether such variations are consistent with the proposed hybridization mechanism would be useful.
Some minor points: Sample preparation details should be added to the Methods section. The scale of features in figures (e.g., Fig. 3a,b) should accurately reflect actual dimensions.
In summary, the manuscript presents careful experiments and a plausible interpretation of the observed inelastic features. At the same time, the current evidence for magnon–phonon hybridization remains largely indirect, and some aspects of the interpretation are not yet fully constrained. Further clarification and strengthening of these points would be important to support the central claim at the level expected for a broad-interest journal such as Nature Communications.

Reviewer #2

(Remarks to the Author)
I thank the authors for carefully discussing my comments, and I can now support its publication.

Version 2:

Reviewer comments:

Reviewer #1

(Remarks to the Author)
The authors have addressed all the concerns and provided convincing responses. I agree to publication in Nat. Commun.

Open Access This Peer Review File is licensed under a Creative Commons Attribution 4.0 International License, which permits use, sharing, adaptation, distribution and reproduction in any medium or format, as long as you give appropriate credit to the original author(s) and the source, provide a link to the Creative Commons license, and indicate if changes were made.
In cases where reviewers are anonymous, credit should be given to 'Anonymous Referee' and the source.
The images or other third party material in this Peer Review File are included in the article’s Creative Commons license, unless indicated otherwise in a credit line to the material. If material is not included in the article’s Creative Commons license and your intended use is not permitted by statutory regulation or exceeds the permitted use, you will need to obtain permission directly from the copyright holder.
To view a copy of this license, visit https://creativecommons.org/licenses/by/4.0/

Pointwise Response to Reviewers' Comments

We thank both reviewers for their constructive comments/suggestions. We provide our pointwise responses to all comments/concerns below in light blue. The changes to the main text (other than trivial typos) are highlighted in blue.

Reviewer #1

In this manuscript, the authors report low-temperature spin-polarized and non–spin-polarized scanning tunnelling spectroscopy (STS) measurements on Fe□GeTe□ (FGT), revealing several discrete inelastic features that they interpret as signatures of magnon–phonon hybridization (magnon polarons). By comparing the observed energies with calculated magnon and phonon dispersions, the authors argue that the peaks correspond to hybrid modes emerging at avoided crossings between acoustic magnons and phonons. Additional magnetic-field and domain-wall–dependent measurements are presented to support the magnonic component of the excitations, and a simplified model for the local density of states is constructed. The topic is timely and the experimental technique is sophisticated. If firmly validated, this work would represent a significant advance. However, at present, several key aspects of the central interpretation remain insufficiently substantiated.

Reply: We thank the reviewer for the comment that "this work would represent a significant advance." We would like to clarify that the key achievement of our model is **not** a modification of "*the local density of states*", which remains essentially unchanged, as shown in Figure 5(b,e). Rather, the central result is that the magnon-phonon hybridization (i.e., magnon polaron) gives rise to a peak in ISTS, as demonstrated in Figure 5 (c,f) and Supplementary Figure 4.

Importantly, our work represents a substantial advance in the field of inelastic electron tunneling spectroscopy itself, as it establishes a microscopic framework for detecting hybrid quasiparticles, specifically magnon polaron, via by ISTS, as illustrated schematically in Figure 1.

Major Comments:

1. The identification of magnon–phonon hybridization is based mainly on energy coincidence with calculated crossings and would benefit from more quantitative support distinguishing hybrid modes from simple magnon–phonon overlap.

Reply: We discussed the difference of hybrid modes (magnetoelastic coupling, $\lambda > 0$) and simple overlap (no magnetoelastic coupling, $\lambda = 0$) and showed it in Figure 5. In the latter case, the ISTS signal is simply the sum of the magnon and phonon component and no ISTS peaks arise in the low energy range, as the DOS of the bosons have no peaks. Only when they hybridize, the selection rules are lifted and peaks in ISTS appear. To avoid the reader missing this result, we now stress this point even more in the revised manuscript.

2. The magnetic-field evolution of the inelastic features is presented qualitatively and requires a more systematic quantitative analysis.

Reply: We thank the reviewer for this suggestion. The drastic changes of the ISTS with even modest fields pinpoint the magnetic nature of these excitations. Showing this has been the

main intention behind presenting the data. The referee is right that we did not present a full analysis in the supplementary information. We have now extended this discussion but a full analysis on a quantitative level would require to describe the dispersions of the bosons in all of the Brillouin zone and especially for magnons, the dispersion is only known along the principal directions. We thus add the discussion of magnon polaron under magnetic field to the Supplementary Information along specific directions, showing how the individual peaks will shift with the magnetic field. We show this data below:

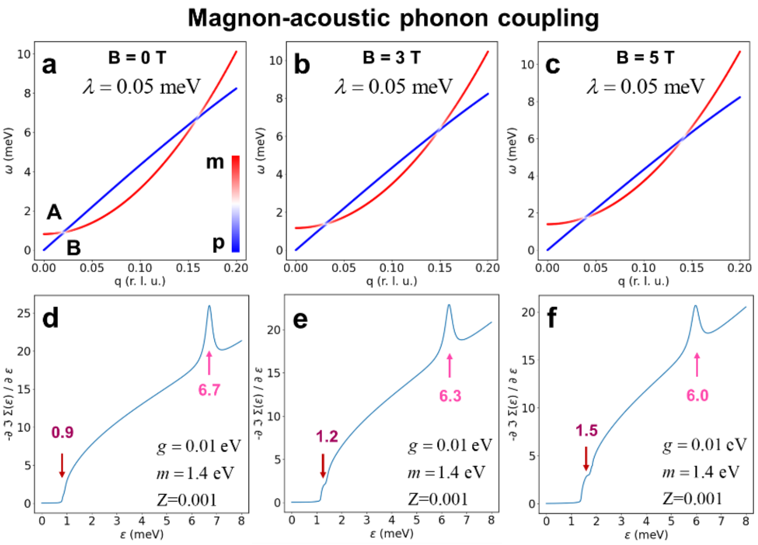

Response Figure 1: *Magnon-acoustic phonon coupling under an applied magnetic field. For magnon-acoustic phonon coupling, there are two avoided band crossings. When the energies of these two avoided band crossings are far away from each other, the increasing magnetic field lifts the magnon dispersion making the energy of lower crossing increase while the energy of the higher crossing decrease.*

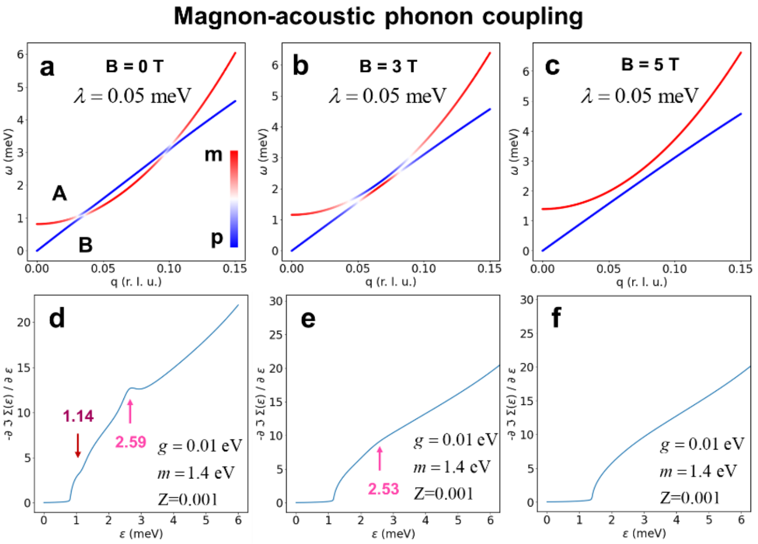

Response Figure 2: *Magnon-acoustic phonon coupling under an applied magnetic field. For magnon-acoustic phonon coupling, there are two avoided band crossings. When the energies of these two avoided band crossings are close with each other, the increasing magnetic field lifts the magnon dispersion driving the emergence and broadening of the two magnon polaron peaks. Eventually, no crossing is observed and a magnon-polaron is not created.*

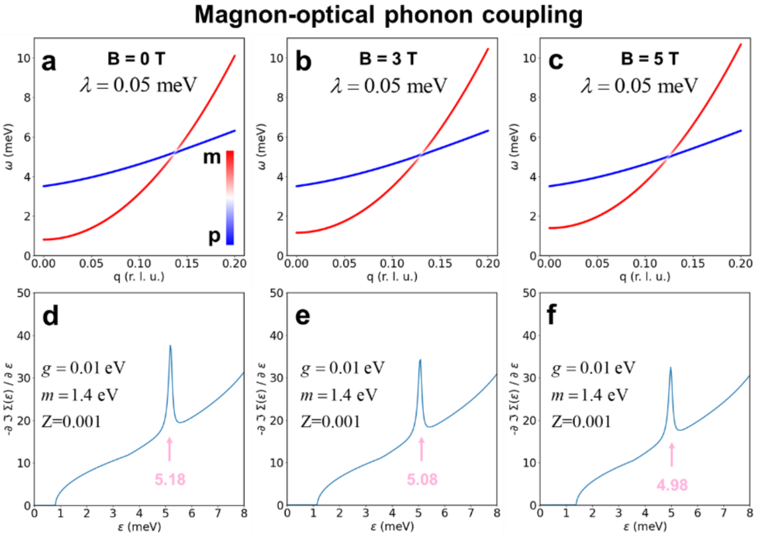

Response Figure 3: *Magnon-optical phonon coupling under an applied magnetic field. For magnon-optical phonon coupling, there is only one avoided band crossing. The increasing magnetic field lifts the magnon band somewhat, whereas the energy of magnon polaron decreases.*

3. The spin-polarized STS contrast across domain walls is modest, and the robustness and reproducibility of the spin-dependent signals need to be better documented.

Reply: We thank the reviewer for this helpful comment. We would like to note that the spin-polarized STS contrast is rather large. We see about 15% in STS at 500mV. We added this information to Figure 3a in the revised manuscript (a reproduction is seen below on the right). This contrast is similar or larger than the contrast reported by Trainer et al., Phys. Rev. B 106, L081405 (2022) (reference [46] in our main text), as shown in Response Figure 4. We would also like to stress that the contrast is very stable. STS maps of FGT including large magnetic domains, skyrmion bubbles and the domain wall profile have been discussed in all detail in our previous work that we also cited (reference [45] in the main text). The contrast in spin-polarized ISTS, i.e. in the inelastic channel, is indeed small, as the ISTS signal is small itself.

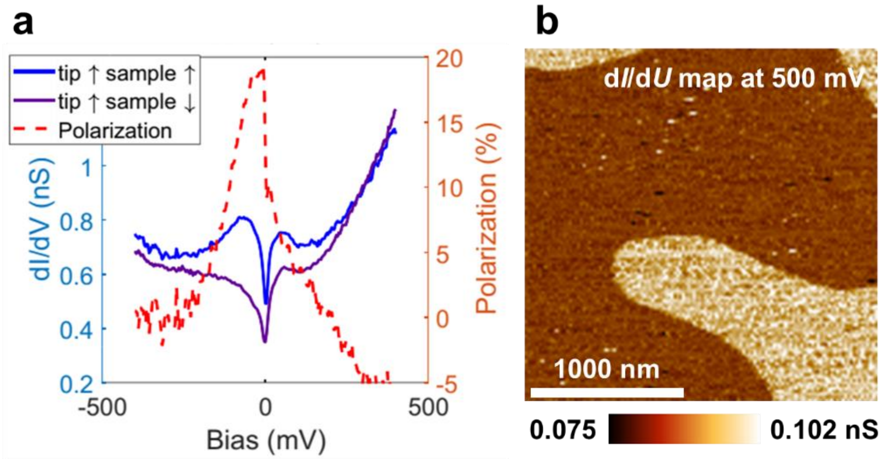

Response Figure 4: ***a*** *Spin-polarized measurements on FGT from Fig. 3f in Phys. Rev. B 106, L081405 (2022) (reference [46] in our main text). It shows a spin polarization of -5% around 400 mV.* ***b*** *Our spin contrast has a spin polarization of 15%.*

4. The applicability of the adopted magnon dispersion to Fe-deficient FGT samples requires stronger justification.

Reply: We thank the reviewer for this helpful comment. All reported Fe_3GeTe_2 (FGT) samples exhibit some degree of Fe deficiency. As we mentioned in the Experimental details of the Methods section, our single crystals $Fe_{2.96}GeTe_2$ have a very low deficiency with only 1.3%. Its Curie temperature T_C reaches 205 K, comparable to the upper range of values reported for FGT in the literature, as mentioned in the Experimental details. We used the best data available in the literature from state-of-the-art Neutron diffraction of $Fe_{2.72}GeTe_2$ samples with a deficiency of 9% and a T_C of 160 K (Reference [14] in the main text), which currently provides the most reliable and quantitative information on the magnetic excitation spectrum of FGT. Thus, the situation is opposite to what the referee suggests. Our samples are of higher quality than those of the neutron diffraction experiments. We now stress this in the manuscript. In the meantime, there is a new publication on magnon dispersion of $Fe_{2.92}GeTe_2$ near the Gamma point, that we now also include (Beier et al., Phys. Rev. B 112, 214414 (2025)). The magnon gap of 0.70 meV agrees well with the value we use of 0.8 meV. All this supports the use of the applicability of the experimental magnon dispersion relation to our work.

5. A clearer distinction between hybrid excitations and purely phononic van Hove singularities is needed.

Reply: We fully agree with the reviewer. This was actually done in the manuscript and displayed in Figure 4. As can be seen, there are no van-Hove singularities of phonons in the energy range of the ISTS peaks. The lowest van Hove peak of phonons is at around 10 meV (Figure 4a). We now state this even more clearly in the revised manuscript.

Minor Comments

1. The manuscript is rather long and includes extensive theoretical exposition; the authors may consider streamlining some sections for clarity.

Reply: The reviewer is right that the theory part is rather extensive. We felt that the complex physics needs extended explanations. In the revised manuscript, we tried to be a more concise and moved part of the calculation to the supplementary. We, however, want to stress that exactly this discussion about self-energy corrections in hybrid modes is one of the important findings of our work.

2. Some figures would benefit from clearer labeling (e.g., consistent color coding between experiment and theory).

Reply: We thank the reviewer for this advice and modify the labeling of figures accordingly.

3. Please clarify whether the use of "spin gap," "Stoner gap," and "magnon gap" refers to the same quantity or to distinct physical concepts.

Reply: We thank the reviewer for this comment. The Stoner gap is the gap in energy and momentum space of single particle spin flip excitations and is not identical to the magnon gap, which is the gap in many particle excitations. We took care in the revised manuscript to define the terms.

4. The temperature stability during long spectroscopy measurements should be stated explicitly.

Reply: We thank the reviewer for this comment. We now added a statement on the temperature stability. Variations are below 3 mK.

5. In several places, the error bars or uncertainties of peak positions are not indicated.

Reply: We thank the reviewer for this very helpful comment and added error bars to the experimental data. The peak positions were not fitted, as the line shape of the inelastic peaks is not known, i.e. there are no statistical error bars for their positions. Instead, we can give an instrumental resolution, which is about $0.6U_{mod}$, that gives practical limits the energy position of the observed maxima and minima. We now mention this in the revised text.

Reviewer #2 :

The manuscript presents a combination of experimental ISTS data using unpolarized and spin-polarized tips, and theoretical modeling to demonstrate magnon-phonon hybridization in Fe3GeTe2. The concept of exploring hybrid excitations in a metallic van der Waals ferromagnet is innovative and aligns with the scope of Nature Communications. However, the evidence presented does not meet the journal's high standards, primarily due to limitations in data depth, methodological rigor, and interpretive robustness. Below, I outline specific concerns that preclude acceptance in its current form.

(1) The ISTS spectra in Fig. 2(d) reveal several distinct peaks (0.3, 0.8, 2.5, 3.8, and 5.2 meV), which are interpreted as signatures of magnon-polarons. However, the data rely on a relatively small dataset (averaged over 121 curves in a 4×4 nm2 area). This raises questions about statistical significance and reproducibility. Claims of this importance require broader spatial mapping and replication across multiple samples to rule out localized artifacts.

Reply: The data of Figure 2d (Fig. 2c in the revised version) is not the only data presented on the inelastic peaks. Figure 2d (Fig. 2c in the revised version) has another 41 spectra with the same energy resolution in color code illustrating the lateral variation over a much larger distance of 20 nm (see Fig. 2d in the revised version). We also presented ISTS as function of temperature and magnetic field in the supplementary information, and finally ISTS data with spin polarized tips consisting of over 91 spectra and extensions of 60 nm, all to illustrate the significance and reproducibility. In fact, we have plenty of data that all showing these peaks. We observed these with all tips we ever tried over extended measuring phases more than two years. Alone the data presented in the paper contains experiments with 7 different tips, unpolarized and spin polarized, over 5 samples. We now state this in the supplementary information. The homogeneity of the observed ITS signal demonstrates the high quality and low Fe deficiency of our samples.

(2) The large-scale topography in Fig. 2(a) indicates an unstable tip condition, likely due to surface contamination. Tip instability can introduce artificial features in STS measurements, potentially explaining the multiple peaks observed in ISTS. Such artifacts might also exhibit domain-wall dependence, as seen in the dI/dV maps. The authors should repeat measurements with a cleaner sample and more stable tip conditions to ensure reliability.

Reply: We thank the reviewer for this insightful comment on the tip induced effect. As mentioned in the above reply, our data were collected over more than two years, rather than performed in a one-time measurement. Our consistent results (normal tip Figure 2(c,d), spin-polarized tip Figure 3, magnetic field dependence Supplementary Figure 1, and temperature

dependence Supplementary Figure 2) with different tips can safely exclude tip induced artificial features in ISTS. Further, note that artificial features would not result in symmetric peak-dip pairs in ISTS. Also, tip features cannot explain the behavior under magnetic field. Similarly, tip features cannot systematically and smoothly change in a way to mimic a magnetic domain wall. We scanned many times back and forth across the domain wall and also the wall profile agrees with simpler spin-polarized STS. We added the discussion of tip instability that could be excluded in our experiments in the main text. The noise in the topography of Fig. 2(a) is actually mostly due to the fact, that our instrument is located in the cellar just below the main entrance of the physics building. We sometimes suffer from minor vibrations, when the door is slammed. Moreover, the sample is very clean. It was cleaved in UHV and was immediately transferred into the STM. Single adsorbents would result into height differences of the order of 100pm, which are absent.

(3) The magnetic-field-dependent ISTS data (Supplementary Fig. 1) are limited to three field values (0T, 3T, 5T), with no clear trend established between peaks across fields. The relationship between peaks at 0 T and those under higher fields is ambiguous. Additionally, the absence of field-dependent ISTS using spin-polarized tips weakens the magnetic origin argument.

Reply: There is no simple trend in the peak positions, as the Zeeman energy moves the quadratic magnon dispersion in energy resulting is shifts of the peaks in different directions, depending on the dispersion. There are also conditions when the dispersions become tangential giving rise to broader peaks. (Please also see reply to Reviewer #1 Comment 2.) We have added a deeper analysis of the peaks' behavior in the supplementary information. Nevertheless, the spectra react strongly to an external magnetic field excluding phonons as a source for the excitation signals. Only magnetic degrees of freedom couple to magnetic fields. In the literature, two accepted proofs for magnetic excitations have evolved. First, the change of the spectral features with magnetic field, that we demonstrated, and second, the change of the spectral intensities when going from a parallel to an antiparallel orientation of spin-polarization in spin-polarized tunneling experiments, that we also showed. With demonstrating both, we went further than usual papers.

(4) The ISTS peaks obtained with W tips (Fig. 2(d)) appear inconsistent with those from spin-polarized tips (Fig. 3(b)). To validate reliability, the authors should directly compare raw ISTS spectra for both tips (for spin-polarized tip, the data on aligned and anti-aligned domains should be shown respectively) in a single figure.

Reply: We thank the reviewer for this suggestion. The raw spectra recorded with W tips were shown in Figure 2d. The raw spectra of the different domains were already shown in direct comparison in Figure 3b in color code. We added the requested curves to aligned and ant-aligned configuration in the revised Figure 3(d,e). The change of the spectra was already plotted in Figure 3d (revised version Figure 3c). The spin-polarized data was taken with a larger modulation (1 mV), i.e. lower energy resolution, compared to the W tip data (0.5 mV). This was necessary, as the small changes due to spin-polarized tunneling and the mapping across the domain wall require a higher signal to noise ratio. Below, we show a comparison of the spin-polarized and unpolarized spectra, where the latter was corrected for the energy resolution to document the agreement. Response Figure 5b shows the convolution of ISTS in Fig. 2d (Fig. 2c in revised version) with lock-in amplifier modulation amplitude Umod = 1 mV. Compared with Response Figure 5a (Fig. 2c of the revised main text), convolution (gray curve) causes the peaks at 0.3 and 0.8 meV to merge into a single peak at 0.8 meV, shifts the 2.3 meV peak to 2.7 meV, and smears the 3.8 meV peak. The 5.2 meV peak shifts to 6.5 meV. Notably, three peak-dip pairs at 0.8, 2.7 and 6.5 meV can be seen for the difference

of SP-ISTS on bright domain and dark domain (Response Figure 5c), which agrees well with the convoluted ISTS (dashes lines in Response Figure 5). We add the comparison to the main text.

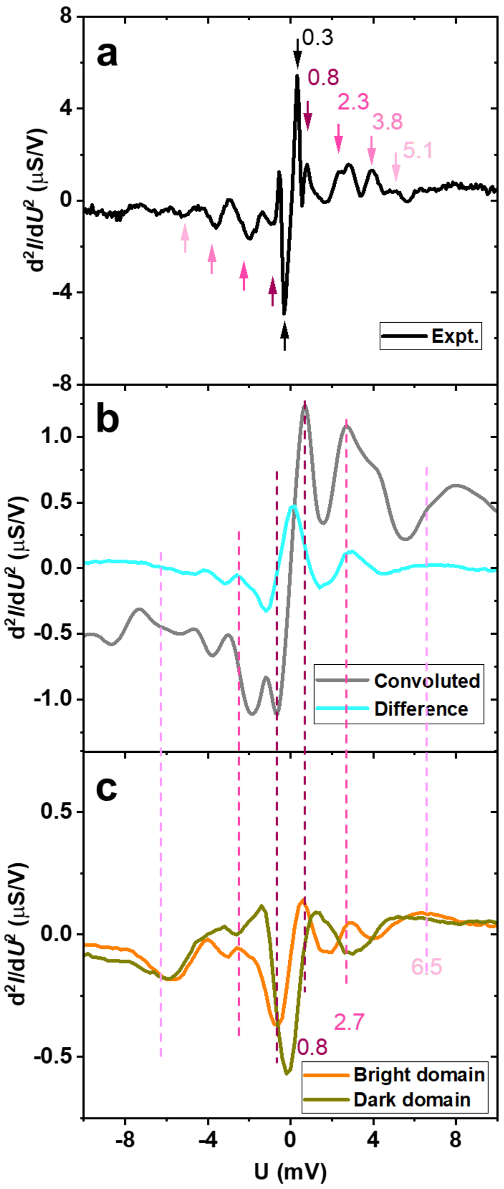

Response Figure 5: *Comparison of spin-polarized ISTS and ISTS with modulation*.

(5) While Fig. 3 claims domain-wall effects in spin-polarized ISTS, the contrast changes are subtle and susceptible to tip-related artifacts. The description of peak-dip asymmetry based on color plots (Fig. 3(b)) is not visually convincing. Raw ISTS spectra should be plotted to clarify these features.

Reply: We thank the reviewer for this suggestion. We added the curves to Figure 3 (see also Reply to (4)). The changes are not susceptible to tip-related artifacts. The transitions between the domains follow the domain wall width and are not abrupt, i.e. not due to tip changes. We added line scans to illustrate this.

(6) The ISTS map at 10 mV (Fig. 3(c)) shows a weak but perceptible transition at the domain wall, potentially due to low signal intensity. The relevance of this transition to the magnon-polaron narrative remains unexplained, undermining its interpretive value.

Reply: We thank the reviewer for this comment. At 10 mV, there is almost no inelastic signal (0 ± 0.04 μS/V) and the contrast is essentially absent within the noise level (see Response Figure 6b), as the referee correctly realizes. With some good will, a weak contrast can be seen. Indeed, there is also a magnon-phonon band crossing around 10 meV as shown in Figure 4(b,c) and with some good will it is also visible as a broad peak in ISTS. Due to its relatively high energy, the excited states become rather short lived and broad which makes them difficult to observe. We therefore restrict the discussion in the paper on signals that are clear, i.e. to the peaks up to about 5 meV. At 5 mV, the contrast is robust with variation about 200% from dark domain (~0.2 μS/V) to bright domain (~0.6 μS/V) (see Response Figure 6a). We discuss this now more precisely and add these average line profiles to Figure 3.

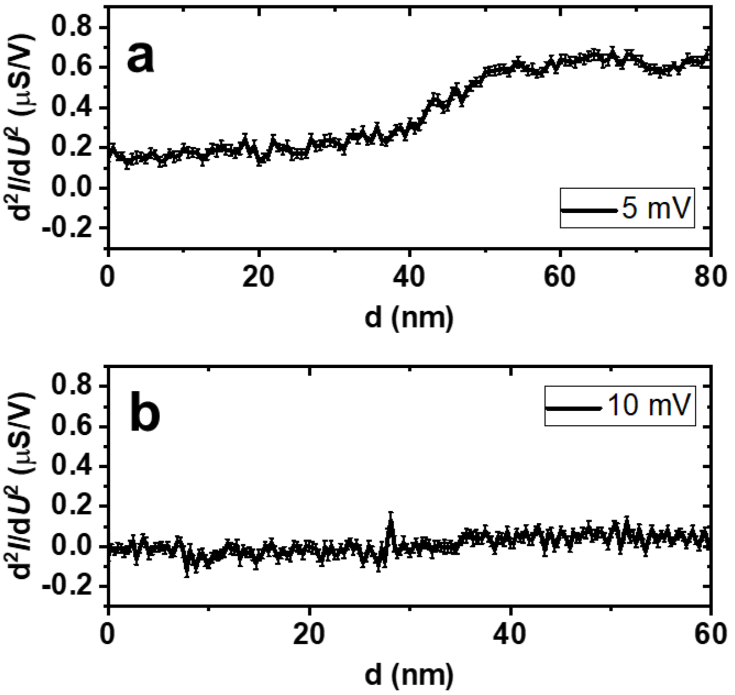

Response Figure 6: *Average line profiles of ISTS maps at 5 mV and 10 mV, respectively.*

(7) The paper omits verification of the spin polarization of Cr-coated tips before and after ISTS measurements.

Reply: We thank the reviewer for this suggestion. The spin polarization of the Cr-coated tips was verified in the manuscript. Prior to each spin-polarized ISTS experiment, we perform spin-polarized STM (SP-STM) imaging to resolve magnetic domain contrast to accurately locate domain walls (Figure 3a). These domain images were acquired immediately before the spin-polarized ISTS measurements and therefore confirm the spin polarization of the tip at the beginning of the experiment.
In addition, the manuscript presents the time evolution of the magnetic contrast during the ISTS measurements as the position d is varied (Figure 3b). Throughout the measurement sequence, the magnetic contrast remains stable, indicating that there is no detectable loss of tip spin polarization during or after the spin-polarized ISTS experiments (no other changes in the ISTS signal than in the area of the smooth transition across the domain wall). After that, we took the spin-polarized ISTS maps shown in Figure 3c (Figure 3f in revised version). Still the domain wall is present indicating spin sensitivity. We can thus safely exclude that the spin-polarization vanished or reversed during the measurements. We added the

verification of the spin polarization of Cr-coated tips during the measurements in the main text.

(8) The claim that "For the Kondo effect to appear, the system needs to obey time-reversal symmetry" is incorrect; Kondo effects can coexist with time-reversal symmetry breaking, as demonstrated in systems like CeRh6Ge4 (Nature 579, 51, 2020). This erroneous premise weakens the argument against alternative interpretations of the dip-like feature in Fe3GeTe2.

Reply: We thank the reviewer for this important clarification. Our original statement was indeed overly simplified and requires refinement. We agree that the Kondo effect can, in principle, coexist with time-reversal symmetry (TRS) breaking, as demonstrated in ferromagnetic or field-polarized Kondo systems, provided that the relevant TRS-breaking energy scales remain smaller than or comparable to the Kondo energy scale.
More specifically, the survival of Kondo screening depends on the competition between the Kondo energy scale $E_K \sim k_B T_K$ and TRS-breaking energy scales such as the Zeeman energy $E_Z \sim g\mu_B B$ or the exchange splitting E_{ex} in a ferromagnetic Kondo lattice. When E_Z or $E_{\mathrm{ex}} \gtrsim E_K$, Kondo screening is strongly suppressed or destroyed. Consistent with this principle, experimentally established ferromagnetic Kondo lattice systems typically satisfy $T_K \gtrsim T_C$, ensuring that Kondo correlations remain robust against magnetic ordering.
In Fe_3GeTe_2, however, the situation is qualitatively different. It displays an exchange splitting on the order of hundreds of meV (ref. [18]), whereas the Kondo temperature claimed in ref. [42] is $T_K \approx 190$ K, yielding $E_K \sim 16$ meV. DFT calculations (i.e. no Kondo physics taken into account) faithfully reproduce experimental band structures and DOS.
As the exchange energy scale in Fe_3GeTe_2 exceeds the suggested Kondo energy scale by more than an order of magnitude, the ground state of FGT is simply ferromagnetic and coherent spin flips of the Kondo screening are energetically forbidden. We revise the discussion of Kondo effect in Fe3GeTe2 in the main text. Finally, our measurements of the magnetization (see Response Figure 8 reproduced from [45]) and resistance (shown in the Supplementary) clearly show an increase in M and a drop of rho with falling temperature, i.e. the opposite behavior expected for a Kondo system.

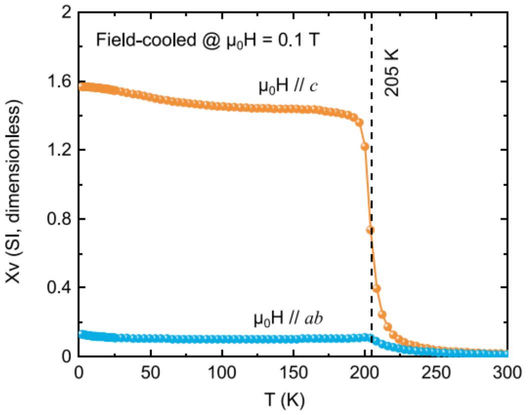

Response Figure 8: Magnetic volume susceptibility (χ_v) as a function temperature measured in field-cooled condition (0.1 T).

(9) The model calculations for electron self-energy and magnon-phonon coupling rely on empirical parameters rather than ab initio-derived values. This simplification fails to capture momentum-dependent hybridization, reducing the quantitative accuracy of predicted band crossings and ISTS peaks.

Reply: We agree with the referee that assessing the magnon-phonon coupling and electronic self-energies from DFT would be highly desirable. However, since no DFT codes

which can tackle this problem consistently exist at this point, this presents a considerable challenge which we are bound to delegate to future studies. Moreover, concerning the specific material that we study, we use the data on the magnon dispersion from inelastic neutron scattering as DFT fails to describe magnonic properties in this itinerant system. For our analysis, the mere presence of the magnon-phonon coupling is sufficient for the demonstration of the effect which results in the peaks in the observed inelastic spectrum due to excitation of a magnon-polaron at the crossings among magnons and phonons bands, while taking into account the k-dependence of the magnon-phonon coupling may renormalize the height of the peaks but is not expected to cause additional features in the inelastic channel away from the crossings, as explained in the manuscript.

(10) The resistivity data (Supplementary Fig. 5) show a kink near 80 K, but the connection to magnon-polaron damping is speculative without direct correlation to ISTS energies or robust theoretical support.

Reply: We agree with the reviewer that the connection is speculative and has been indicated as such. The theoretical support on resistivity is beyond the scope of our manuscript, which is interesting in the future investigation. The effect of carrier scattering in the transport regime with these excitations is not related to that of tunneling. In the latter case, we can choose the energy of the carriers, in the first, we cannot. The figure instead has the purpose to illustrate the absence of an increase of the resistivity due to the Kondo effect. We thus remove the discussion of the kink.

Despite the novel premise, the manuscript lacks the data richness, methodological rigor, and interpretive accuracy required for Nature Communications. The concerns outlined above (particularly regarding data quality and statistical validity) significantly undermine the claims of magnon-polaron observation. I recommend a substantial revision addressing these points, with potential resubmission to a more specialized journal after further validation.

Reply: We thank the reviewer for these helpful comments, which have significantly improved the manuscript. We have revised the manuscript accordingly and are now more confident in the clarity and robustness of the revised version.

In this work, we performed extensive measurements using W tips and Cr-coated W tips, as well as studies of magnetic-field and temperature dependence. These results are presented in the main text and the supplementary information. In addition, we developed a theoretical model that establishes a microscopic framework for detecting hybrid quasiparticles (i.e., magnon polaron) via inelastic scanning tunneling spectroscopy (ISTS). Taking together, our experimental and theoretical results represent a significant advance, as also acknowledged by Reviewer #1. We are not aware of any other study performed at the same level of theoretical and experimental rigor and insight, which unambiguously demonstrates the formation of magnon-polarons in a two-dimensional magnet with STM. We are confident that our work will become a landmark in the field and will serve as an inspiration and reference point for further studies in this area to come. We thus firmly believe that both thematically and technically, our work belongs to the core of Nature Communications scope.

Pointwise Response to Reviewers' Comments

We thank the reviewers for their constructive comments/suggestions. We provide our pointwise responses to all the comments/concerns below in light blue. The changes to the main text are highlighted in blue.

Reviewer #1

The authors have carefully revised the manuscript and addressed several points raised in the previous round. The work presents high-quality ISTS measurements on Fe3GeTe2 and tackles a timely and interesting problem. The comparison between the observed inelastic features and calculated magnon–phonon dispersions is clearly presented, and the interpretation in terms of magnon–phonon hybridization is physically reasonable. However, after re-evaluating the revised manuscript, I feel that the central claim, the identification of magnon–phonon hybrid quasiparticles, is still supported primarily by indirect evidence. While the revision has improved the presentation, several aspects of the interpretation would benefit from further clarification to more firmly establish the proposed picture:

1. The assignment of the observed peaks to magnon–phonon hybrid modes is mainly based on their correspondence with calculated avoided crossings. While this agreement is suggestive, it remains somewhat indirect, and it would be helpful to clarify how these features can be distinguished from a superposition of magnon and phonon excitations.

Reply: Our reasoning regarding the hybridization is based on observables and theory, and is substantiated by several critical tests, that we present. If we understand the reviewer correctly, he/she argues that the peak energies in ISTS and the energies of the avoided level crossings might be a coincidence and might not be related to hybridization. We have now given a more complete explanation regarding the key distinction between a simple coexistence of uncoupled magnon and phonon excitations and genuine hybrid modes formed through magnon–phonon coupling. In the former case, the two excitations retain their separate identities and independent field responses; in the latter, magnon–phonon coupling produces mixed modes with characteristic avoided crossings and modified magnetic-field evolution. Only in the hybridized case, peaks in the ITS spectra are expected when taking the very general condition of magneto-elastic coupling. In case of no hybridization, the ITS signal is a smooth function related to the density of states of the excitations. The experimental observations are not in agreement with the uncoupled scenario, as also the assumption of a vanishing magneto-elastic coupling is at odds with literature.
In the following, we summarized the evidences, we present:

First, the observed excitations exhibit clear magnetic contrast in spin-polarized (see Figure 3) and magnetic-field-dependent measurements (see Supplementary Figure 1), establishing that they cannot not be purely phononic.

Second, they cannot be attributed to bare magnons. In particular, the calculated magnon density of states is featureless below 5 meV (see Figure 4a) and therefore cannot account for the discrete peaks observed in ISTS. Moreover, for uncoupled magnons, all modes are expected to shift monotonically to higher energy with increasing magnetic field. In contrast, our measurements show both upward and downward energy shifts (see Supplementary Fig. 1), which are inconsistent with a purely magnonic origin.

Third, this contrasting field dependence is fully explained by magnon–phonon hybridization. Near the avoided crossing, the hybrid modes inherit mixed character, leading

to nontrivial energy shifts that depend on the avoided band crossings between magnon and phonon (see Supplementary Figs. 2–4 and response to point 2 of the reviewer, just below).

Therefore, the combination of (i) magnetic sensitivity, (ii) inconsistency with bare magnon behavior, and (iii) agreement with hybridization-induced avoided crossings energies and shifts with magnetic field, distinguishes the observed features from a simple superposition of independent magnon and phonon excitations.

2. The magnetic-field-dependent and spin-polarized measurements support a magnonic contribution. A more quantitative comparison with expected magnetic energy scales (e.g., field-induced shifts) would help to assess whether all observed modes consistently follow a magnonic origin.

Reply: We thank the reviewer for this question. We have extended the quantitative comparison between the observed field dependence and the expected magnetic energy scales.

According to the magnon‑phonon hybridization scenario (Supplementary Figs. 2-4), different hybrid branches exhibit distinct magnetic-field responses: the first avoided crossing with acoustic phonons shifts to higher energy with increasing magnetic field, whereas the second avoided crossing with acoustic phonons and the avoided crossing with optical phonons shifting to lower energy.

Experimentally, the energy peak at 0.8 meV shifts upward to 1.1 meV at 3 T and 1.25 meV at 5 T (see Response Fig. 1), in quantitative agreement with the expected Zeeman energy shifts of 0.35 meV (3 T) and 0.58 meV (5 T). This agreement confirms that the low-energy mode follows the expected magnetic scaling and provides a direct calibration of the magnonic energy scale in our measurements.

By contrast, the higher-energy peaks shift downward with increasing magnetic field (see Response Fig. 1). Such opposite field dependence cannot be explained within a bare magnon picture, in which all magnon modes shift monotonically upward with field. Instead, the observations agree with a downward shifts of the hybridized magnon‑phonon, where the field evolution depends on the mixed magnonic and phononic character of each hybrid branch.

We have added this quantitative discussion to Supplementary Information Note I.

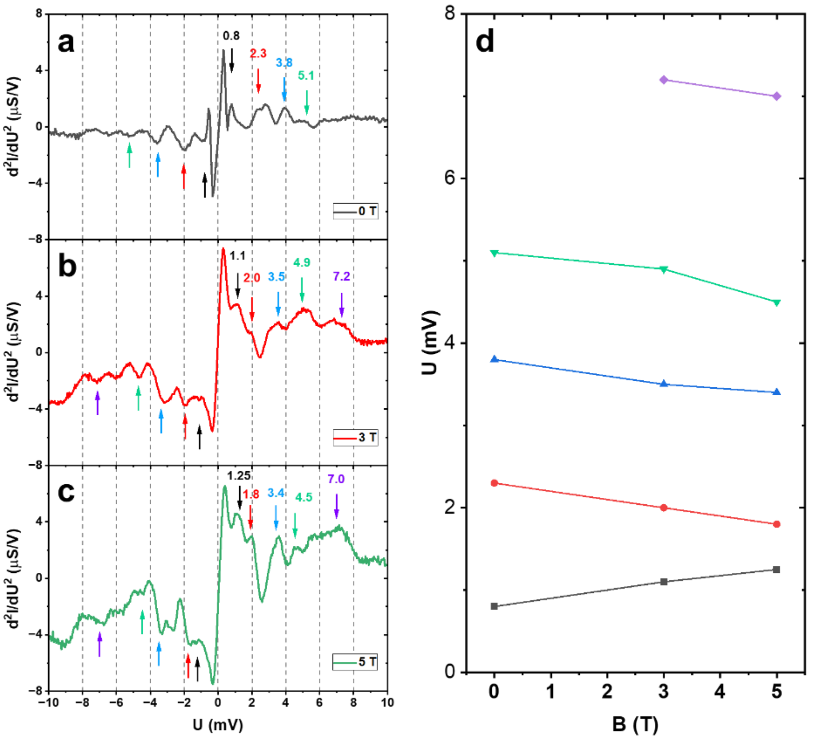

Response Figure 1: *Magnetic field induced energy variations of magnon-phonon hybrid modes.*

3. Although possible alternative origins (e.g., phonon-related features or tip effects) are discussed, it remains unclear whether the current analysis uniquely favors the hybridization scenario over these alternatives. Further clarification would help to reinforce the proposed interpretation.

Reply: We clarify that the hybridization scenario is uniquely supported by a combination of experimental observations and microscopic model calculations, which are not simultaneously explained by alternative mechanisms, i.e. phonon energies do not shift with magnetic field, phonons are insensitive to the spin, lateral changes of the spectrum across a magnetic domain wall cannot be due to the tip, etc. In more detail:

First, the observed excitations exhibit clear magnetic contrast in spin-polarized measurements, establishing their magnetic character and ruling out a purely phononic or tip-related origin. At the same time, their energies coincide with the expected magnon–phonon band crossing, indicating coupling to lattice degrees of freedom. This simultaneous presence of magnetic and phononic characteristics is a defining signature of magnon polarons.

Second, the energies of the observed features and their shifts with magnetic field quantitatively match the calculated magnon–phonon crossing points. Such agreement is highly nontrivial and cannot be simultaneously reproduced by alternative mechanisms without invoking fine-tuned (and magnetic field dependent parameters) coincidence, especially given the systematic field and momentum dependence.

Third, magnon–phonon coupling must lead to avoided band crossings, which naturally produce pronounced features in ISTS as shown by our self-energy calculations. Note that magnon-phonon coupling has been reported in Fe_3GeTe_2 both theoretical and experimental (see Refs. [18, 59-61] in the main text). In contrast, alternative scenarios—such as purely phononic modes, electronic inelastic channels, or tip-induced effects—do not reproduce the observed combination of energy position, magnetic sensitivity, and spectral lineshape.

Taken together, only the hybridization scenario consistently accounts for the combined constraints of magnetic sensitivity, energy position, field dependence, and spectral structure. We have clarified this reasoning in the revised manuscript.

4. The reported spectra show local reproducibility; however, the extent to which these features are spatially uniform beyond the measured regions remains unclear. Additional discussion of spatial consistency would help establish the intrinsic nature of the observed excitations.

Reply: The local spatial consistency of the observed excitations is explicitly demonstrated in Figs. 2 and 3. In Fig. 2, the spectra are reproducible over a lateral extent of ~20 nm, while Fig. 3 extends this range to ~60 nm. Importantly, these two measurements and the measurements on the filed dependence and temperature dependence (Supplementary Figures 1 and 5) were performed at different positions, partly even on different samples and with different tips, yielding consistent spectral features not only on local scale but on long scales and different samples and tips, i.e. the reproducibility, that the referee asks for. We even more stress this point in the revised manuscript by explicitly discussing the spatial uniformity and its implication for the intrinsic nature of the excitations.

5. The inelastic peaks exhibit noticeable differences in intensity, including a relatively strong low-energy feature. Further discussion on whether such variations are consistent with the proposed hybridization mechanism would be useful.

Reply: We thank the reviewer for this question. The variation in peak intensity of the different peaks in one spectrum is indeed consistent with the hybridization scenario and arises from lifetime effects. The ISTS intensity reflects the bosonic spectral function weighted by the tunneling matrix elements. In the hybridization regime, the spectral weight is redistributed between magnonic and phononic components, while the quasiparticle lifetime decreases with increasing energy due to additional decay channels in accord with Fermi liquid theory. This combined effect naturally leads to a stronger low-energy feature and reduced high-energy intensity. This effect has been seen in many ISTS studies. We now discuss this in the revised manuscript. Therefore, the observed intensity variation reflects the energy-dependent lifetime, and is fully consistent with the proposed magnon－phonon hybridization mechanism.

Some minor points: Sample preparation details should be added to the Methods section. The scale of features in figures (e.g., Fig. 3a,b) should accurately reflect actual dimensions.

Reply: We thank the reviewer for these suggestions and extended the Method section correspondingly.

In summary, the manuscript presents careful experiments and a plausible interpretation of the observed inelastic features. At the same time, the current evidence for magnon–phonon hybridization remains largely indirect, and some aspects of the interpretation are not yet fully constrained. Further clarification and strengthening of these points would be important to support the central claim at the level expected for a broad-interest journal such as Nature Communications.

Reply: We thank the reviewer for this important assessment. In response, we have taken several steps to clarify and strengthen the evidence supporting magnon－phonon hybridization, as discussed point by point, above.

The revised manuscript highlights the level of support in line with the expectations for a broad-interest journal such as Nature Communications.
